# The association of diet carbohydrates consumption with cognitive function among US older adults modification by daily fasting duration

**DOI:** 10.3389/fnagi.2022.991007

**Published:** 2022-09-26

**Authors:** Shengnan Zhao, Tianshu Han, Xinyi Pei, Yuhua Song, Yuntao Zhang, Lin Liu, Xuanyang Wang, Wanying Hou, Changhao Sun

**Affiliations:** National Key Discipline, Department of Nutrition and Food Hygiene, School of Public Health, Harbin Medical University, Harbin, China

**Keywords:** carbohydrate, daily fasting duration, cognitive function, older adults, NHANES

## Abstract

Dietary carbohydrate consumption was related to cognitive function. Whereas, there was no study investigate the association of dietary carbohydrate consumption with cognitive function modification by daily fasting duration. This study aims to examine the association between dietary carbohydrate consumption and cognitive function among participants with different daily fasting duration. In this cross-sectional study, 2485 adults aged over 60 years from the nationally representative data of the National Health and Nutrition Examination Survey (NHANES, 2011–2014) were enrolled. Percentage energy from carbohydrates was present in both quartiles and continuous forms. Daily fasting duration = 24 – (timing for dinner – breakfast). Cognitive function was assessed by the Consortium to Establish a Registry for Alzheimer’s Disease Word List Learning (CERAD-WL), CERAD Word List Delayed Recall (CERAD-DR), Animal Fluency (AF), and Digit Symbol Substitution (DSST) Test. Multiple logistic regression and linear regression models were developed to examine the association of dietary carbohydrates with cognitive function among participants with different daily fasting duration. Restricted cubic spline models were also applied. Compared with the lowest quartile of percentage energy from carbohydrates, the highest quartile had higher ORs of poor cognitive performance among total participants [(ORCERAD-WL 1.84 95% CI 1.25–2.71); (ORCERAD-DR 1.45 95% CI 1.10–1.91)] and participants with daily fasting duration fewer than 16 h [(ORCERAD-WL 2.14 95% CI 1.29–3.55); (ORCERAD-DR 1.51 95% CI 1.05–2.17)] but not in participants with daily fasting duration of more than 16 h. Further, the negative associations between percentage energy from carbohydrates and CERAD-WL score were still significant in addition to participants whose daily fasting duration was more than 16 h. Additionally, dose-response associations were detected between dietary carbohydrates and cognitive decline, while “U” curves were observed among participants whose daily fasting duration was more than 16 h. This study indicated that dietary carbohydrates consumption was associated with poor cognitive performance, but not in participants whose daily fasting duration was more than 16 h among US older adults. The current analysis provides evidence that a longer daily fasting duration may improve the harmful effect of dietary carbohydrates on cognitive function.

## Introduction

With the increasing aging population, the incidence of age-related cognitive decline that interferes with daily life is increasing as well as being a major public health challenge for the U.S (Hebert et al., 2013; Rajaram et al., 2019). The progression from cognitive decline to dementia is continuous and irreversible, and there is currently no effective drug treatment for dementia (Dong et al., 2020). Further, there is a growing body of evidence indicating that nutrition plays an important role in the prevention of cognitive decline and dementia (Srisuwan, 2013; Gill and Seitz, 2015).

Emerging evidence has indicated that the percentage of carbohydrates in a meal has an acute impact on cognitive function (Dye et al., 2000; Leigh Gibson and Green, 2002; Hoyland et al., 2008). A large body of literature reported the harmful impacts of carbohydrate consumption on cognitive function (Ye et al., 2011; Roberts et al., 2012; Taylor et al., 2017). Further, it appeared that abnormal glucose-insulin regulation can lead to inflammation and oxidative stress in the brain (Rains and Jain, 2011; Sripetchwandee et al., 2018), which contribute to cognitive function decline, dementia, and neurodegeneration (Pugazhenthi et al., 2017). Although dietary carbohydrate consumption influences metabolic and cognitive function across the whole life course, a growing number of findings indicated that the influences of meal-timing on cognitive function may be as large as the meal itself (Mattson et al., 2014; Currenti et al., 2021; Davis et al., 2021). Recent studies have indicated that altering meal-timing could improve cognitive function (Gupta et al., 2019; Davis et al., 2021). Especially, intermittent fasting was considered to be an efficient manner for the prevention of cognitive decline and dementia (Balasubramanian et al., 2020). However, there was no study examined the association between diet carbohydrates consumption and cognitive function by considering daily fasting duration simultaneously. Therefore, we hypothesized that the association between diet carbohydrates consumption and cognitive function could be modified by daily fasting duration, which meant that people with high carbohydrate consumption may improve their cognitive function by regulating their daily fasting duration.

In total, to examine this hypothesis, this study aims to invested the association of diet carbohydrates consumption with cognitive function among participants with a daily fasting duration of more or fewer than 16 h among the US National Health and Nutrition Examination Survey (NHANES, 2011–2014) older adults.

## Materials and methods

### Study population

The NHANES is a nationally representative, continuous cross-sectional study of the population in the United States, which is a complex, stratified, multistage probability cluster sampling design. Detailed descriptions of NHANES were provided elsewhere (Shan et al., 2019). This study involved the data of older adult participants of NHANES (2011–2014) aged over 60 years, who provided dietary data of carbohydrates and energy, the timing for breakfast and dinner, and completed the cognitive function test. Participants with extremely energy consumption (<500 kcal/day or >3,500 kcal/day for women; <800 kcal/day or >4,200 kcal/day for men) were excluded. Overall, a total of 2485 participants were included. A flow chart of the screening process for the selection of eligible participants was shown in Supplementary Figure 1. The institutional review board approval of the National Center for Health Statistics and written informed consent were obtained before data collection.

### Measurement of dietary data and daily fasting duration

Participants’ food intakes for two non-consecutive days were collected through 24-h dietary recall interviews. The first 24-h dietary recall was conducted in person, and the second one was conducted 3–10 days later via telephone. Dietary carbohydrates and energy consumption were estimated with the USDA’s Food and Nutrient Database for Dietary Studies (FNDDS), and the mean values of energy and carbohydrates consumption for day one and day two of the 24 h dietary recall were used in analyses. Meal-timing for breakfast and dinner were participants’ self-reported.

Percentage energy from carbohydrates (%) and daily fasting duration were calculated as follows: Percentage energy from carbohydrates (%) = carbohydrates (g/day) * 4 (kcal/g)/energy (kcal/day); Daily fasting duration = 24 – (timing for dinner – timing for breakfast).

### Cognitive function

A series of cognitive tests were performed in the NHANES (2011–2014) survey, which was among participants aged over 60 years. Cognitive function was collected by a Mobile Examination Center (MEC) and was evaluated by the Consortium to Establish a Registry for Alzheimer’s Disease Word List Learning Test (CERAD-WL), the CERAD Word List Delayed Recall Test (CERAD-DR), the Animal Fluency (AF) test and the Digit Symbol Substitution Test (DSST), which have been widely used in epidemiological and clinical studies (Canning et al., 2004; Fillenbaum et al., 2008; Clark et al., 2009; Gao et al., 2009; Qin et al., 2017; Jaeger, 2018). The CERAD test consisted of three consecutive learning trials (CERAD-WL) and a delayed recall trial (CERAD-DR), in which a total score across three learning trials range from 0 to 30 and a delayed recall trial ranged from 0 to 10, respectively (Morris et al., 1989). In the CERAD-WL trails, participants were asked to read 10 unrelated words, one at a time. In the CERAD-DR trial, participants were asked to recall as many words as possible after the AF and DSST tests (Dong et al., 2020). Animal Fluency Test was a part of executive function, and participants were asked to name as many animals as possible within a minute to examine categorical verbal fluency. The score is the sum of the number of correct answers ranging from 3 to 39 (Dong et al., 2020). The DSST test was performed to evaluate processing speed, sustained attention, and working memory, which is a performance module of the Wechsler Adult Intelligence Scale. The score for DSST ranged from 0 to 133 (Dong et al., 2020). The higher the score, the better the cognitive function on all the above tests.

Furthermore, there is no gold standard for the CERAD Word List Learning, CERAD Word List Recall, Animal Fluency, and DSST tests to determine low cognitive performance. Therefore, according to the published research, the 25th percentile of the score, the lowest quartile was used as the cutoff point to determine low cognitive performance in the current study (Chen et al., 2017). In this study, a total of three groups of participants were obtained, including the total participants, participants whose daily fasting duration was fewer than 16 h, and participants whose daily fasting duration was more than 16 h. For the CERAD Word List Learning test, the cutoff point values of 16, 15, and 16 were used to determine the low cognitive function of the three groups of participants, respectively. For the CERAD Word List Recall, the cutoff point values of 5, 4, and 5 were used to determine the low cognitive function of the three groups of participants, respectively. For the Animal Fluency Test, the cutoff point values of 13, 12, and 13 were used to determine the low cognitive function of the three groups of participants, respectively. For the DSST test, the cutoff point values of 34, 29, and 36 were used to determine the low cognitive function of the three groups of participants, respectively.

### Covariates

Potential covariates included age (years old), sex (male/female), race/ethnicity (non-Hispanic white/non-Hispanic black/Mexican American/other), education level (< Grade 9/Grade 9–11/high school graduate/GED or equivalent/some college or Associate degree/college graduate or above), annual household income (< $20,000/$20,000 – $45,000/$45,000 – $75,000/ > $100,000), regular exercise (yes/no), current smoker (yes/no), current drinker (yes/no), supplements use (yes/no), Body Mass Index (Kg/m^2^), total intake of energy (kcal/day), carbohydrates (g/day), dietary fiber (g/day), diet quality calculated by the Alternative Healthy Eating Index (AHEI) (Wang et al., 2014), self-reported of diabetes, hypertension, cardiovascular diseases (ever diagnosed with congestive heart failure, coronary heart disease, angina, heart attack, and stroke), cancer (ever diagnosed with cancer), and sleep disorders (ever diagnosed with sleep disorders).

### Statistical analyses

All statistical analyses were performed by R 3.6.2. The new sample weight was calculated according to the NHANES analytical guidelines (the 2-year sample weight divided by 2) (National Health and Nutrition Examination Survey, 0000). Demographic characteristics, dietary consumption, and anthropometric measurements across quartile of percentage energy from carbohydrates (%) were presented as weighted means (95% CIs) for continuous variables and weighted percentages (95% CIs) for categorical variables. *P* values were calculated by general linear models for continuous variables adjusting for age and the chi-squared test for categorical variables across quartile of percentage energy from carbohydrates (%).

Multiple logistic regression models were developed to examine the association of dietary carbohydrates with poor cognitive performance among total participants, participants whose daily fasting duration was more than 16 h, and participants whose daily fasting duration was fewer than 16 h, respectively. Odds ratios (ORs) and 95% confidence intervals (CIs) were provided. Categorical variables were modeled as continuous variables through the assignment of the median value to each quartile to test linear trends. Model 1 was adjusted for age, sex, and ethnicity; Model 2 was additionally adjusted for income, education, exercise, current smoker, current drinker, supplement use, BMI, total intake of energy, AHEI, and dietary fiber intake; Model 3 was additionally adjusted for self-reported of diabetes, hypertension, cardiovascular diseases (congestive heart failure, coronary heart disease, angina, heart attack, and stroke), cancer, and sleep disorders.

Linear regression models were performed to examine the association of dietary carbohydrates with the score of cognitive tests among the three groups of participants. β and 95% CIs were provided. Categorical variables were modeled as continuous variables through the assignment of the median value to each quartile to test linear trends.

Additionally, restricted cubic splines were performed to examine the dose-response associations in the logistic regression Model 3 with three knots located at the 5th, 50th, and 95th percentiles of percentage energy from carbohydrates (%) among the three groups of participants.

All analyses (excluding restricted cubic splines) in the current study incorporated sample weights, stratification, and clustering of the complex sampling design to ensure the nationally representative estimates according to NHANES analytic guidelines. A two-sided *P* < 0.05 was considered to be statistically significant.

### Sensitivity analyses

Four sensitivity analyses were carried out between dietary carbohydrates and poor cognitive performance among total participants, participants whose daily fasting duration was more than 16 h, and participants whose daily fasting duration was fewer than 16 h: (1) excluding participants with sleep disorders, which was a traditional risk factor of cognitive function (Irwin and Vitiello, 2019); (2) further adjusted with timing for breakfast and dinner; (3) examining the association between dietary carbohydrates consumption (g/day) and poor cognitive performance; (4) examining the association between dietary carbohydrates and poor cognitive performance defined by total score of CERAD-WL, CERAD-DR, DSST, and AF test. We also performed 3 sensitive analyses among participants 1) male and female; 2) Non-Hispanic Black, Non-Hispanic White, and other; 3) regular exercise and non-regular exercise.

## Results

### Characteristics of participants

Supplementary Table 1 illustrates the characteristics among total participants aged over 60 years across the quartile of percentage energy from carbohydrates (%) from NHANES 2011–2014 in this study (*N* = 2485). Among the 2485 participants, the percentage of participants with poor cognitive performance including CERAD-WL, AF, and DSST showed increasing trends in quartile 4 of percentage energy from carbohydrates (%) compared with quartile 1. Compared with participants in quartile 1, there were more older participants in quartile 4. Compared with participants in quartile 1, participants in quartile 4 were more likely to have less annual household income, and lower education levels.

### Association of dietary carbohydrates with poor cognitive performance among total participants, participants whose daily fasting duration was more or fewer than 16 h

The association of dietary carbohydrates with poor cognitive performance among total participants is shown in Table 1. Compared with participants in quartile 1 of percentage energy from carbohydrates, those in quartile 4 had a higher OR with a 95% confidence interval (95% CI) for the CERAD-WL test (OR Model1 2.09 95% CI 1.55–2.81) when adjusted for age, gender, and ethnicity in Model 1. When further adjusted for other demographic, dietary, and disease information, participants in quartile 4 of percentage energy from carbohydrates were still associated with the CERAD-WL test in Model 2 and Model 3 (ORModel2 1.87 95% CI 1.28–2.73); (ORModel3 1.84 95% CI 1.25–2.71). Similarly, participants in the highest quartile of percentage energy from carbohydrates were associated with the CERAD-DR test in all Models (ORModel1 1.60 95% CI 1.23–2.08); (ORModel2 1.44 95% CI 1.10–1.90); (ORModel3 1.45 95% CI 1.10–1.91). Further, participants in quartile 4 of percentage energy from carbohydrates were associated with the AF test (ORModel1 1.44 95% CI 1.09–1.90) and DSST test (ORModel1 1.82 95% CI 1.28–2.58) when adjusted for age, gender, and ethnicity in Model 1. No association was observed when additionally adjusted for other demographic, dietary, and disease information in Model 2 and Model 3. When deciles of percentage energy were shown in continuous form, the OR of CERAD-WL per 10% increase of percentage energy from carbohydrates was (OR 1.09 95% CI 1.04–1.13) in fully adjusted models.

**TABLE 1:** Association of dietary carbohydrates with poor cognitive performance among total participants.

Cognitive test	Quartiles of percentage energy from carbohydrates				*P*_*for trend*_	*P*_*for interaction with fasting duration*_	Per 10% increase of percentage energy from carbohydrates	*P*-value
	Q1 (*N* = 621)	Q2 (*N* = 621)	Q3 (*N* = 621)	Q4 (*N* = 622)				
**CERAD-WL**								
Case/N	135/621	154/621	177/621	209/622				
Model 1 OR (95% CI)	1.00 (Reference)	1.07 (0.73–1.57)	1.20 (0.99–1.47)	2.09 (1.55–2.81)	<0.001	0.457	1.10 (1.06–1.14)	<0.001
Model 2 OR (95% CI)	1.00 (Reference)	1.18 (0.80–1.74)	1.18 (0.93–1.50)	1.87 (1.28–2.73)	0.004	0.886	1.08 (1.04–1.13)	0.002
Model 3 OR (95% CI)	1.00 (Reference)	1.15 (0.77–1.73)	1.16 (0.91–1.46)	1.84 (1.25–2.71)	0.007	0.774	1.09 (1.04–1.13)	0.003
**CERAD-DR**								
Case/N	213/621	224/621	264/621	272/622				
Model 1 OR (95% CI)	1.00 (Reference)	1.04 (0.75–1.44)	1.18 (0.90–1.55)	1.60 (1.23–2.08)	0.002	0.185	1.05 (1.02–1.09)	0.009
Model 2 OR (95% CI)	1.00 (Reference)	1.09 (0.80–1.50)	1.17 (0.90–1.53)	1.44 (1.10–1.90)	0.018	0.370	1.03 (0.99–1.07)	0.101
Model 3 OR (95% CI)	1.00 (Reference)	1.09 (0.78–1.51)	1.19 (0.91–1.55)	1.45 (1.10–1.91)	0.019	0.334	1.03 (0.99–1.07)	0.098
**AF**								
Case/N	145/621	175/621	190/621	219/622				
Model 1 OR (95% CI)	1.00 (Reference)	0.95 (0.62–1.45)	1.07 (0.81–1.42)	1.44 (1.09–1.90)	0.003	0.006	1.05 (1.02–1.07)	0.001
Model 2 OR (95% CI)	1.00 (Reference)	1.05 (0.63–1.75)	1.06 (0.73–1.53)	1.20 (0.80–1.82)	0.339	0.069	1.02 (0.98–1.06)	0.382
Model 3 OR (95% CI)	1.00 (Reference)	1.03 (0.61–1.74)	1.05 (0.73–1.51)	1.18 (0.77–1.81)	0.403	0.048	1.02 (0.98–1.06)	0.441
**DSST**								
Case/N	123/621	131/621	165/621	204/622				
Model 1 OR (95% CI)	1.00 (Reference)	0.94 (0.69–1.30)	1.33 (0.97–1.84)	1.82 (1.28–2.59)	< 0.001	0.010	1.09 (1.05–1.14)	< 0.001
Model 2 OR (95% CI)	1.00 (Reference)	1.12 (0.79–1.59)	1.24 (0.88–1.76)	1.26 (0.86–1.85)	0.197	0.055	1.03 (0.99–1.08)	0.148
Model 3 OR (95% CI)	1.00 (Reference)	1.07 (0.75–1.54)	1.19 (0.84–1.70)	1.22 (0.82–1.83)	0.278	0.042	1.03 (0.98–1.08)	0.225

Model 1: adjusted for age, gender, ethnicity.

Model 2: additionally adjusted for income, education, exercise, current smoker, current drinker, supplement use, BMI, total intake of energy, AHEI, dietary fiber intake.

Model 3: additionally adjusted for self-reported of diabetes, hypertension, cardiovascular diseases (congestive heart failure, coronary heart disease, angina, heart attack, and stroke), cancer, and sleep disorders.

BMI, body mass index; AHEI, alternative healthy eating index; Q, quartile; CERAD-WL, Consortium to Establish a Registry for Alzheimer’s Disease Word List Learning Test; CERAD-DR, CERAD Word List Delayed Recall Test; AF, Animal Fluency; DSST, Digit Symbol Substitution Test.

The association of percentage energy from carbohydrates (%) with poor cognitive performance among participants whose daily fasting duration was fewer than 16 h is shown in Table 2. Similar to the association among all participants, participants in the highest quartile of percentage energy from carbohydrates were significantly associated with the CERAD-WL test (OR Model1 2.37 95% CI 1.62–3.46); (OR Model2 2.19 95% CI 1.35–3.56); (OR Model3 2.14 95% CI 1.29–3.55) as well as CERAD-DR test (OR Model1 1.66 95% CI 1.16–2.38); (OR Model2 1.51 95% CI 1.05–2.19); (OR Model3 1.51 95% CI 1.05–2.17) compared with quartile 1. For the AF test, participants in the highest quartile of percentage energy from carbohydrates have a higher OR (OR 1.38 95% CI 1.01–1.90) when adjusted for age, gender, and ethnicity in Model 1. No association was observed when further adjusted for other demographic, dietary, and disease information in Model 2 and Model 3. For the DSST test, participants in quartile 4 of percentage energy from carbohydrates had higher ORs (OR Model1 1.94 95% CI 1.39–2.71) (OR Model2 1.52 95% CI 1.05–2.19) (OR Model3 1.44 95% CI 1.00–2.11) in fully adjusted models. When deciles of percentage energy were shown in continuous form, the OR of CERAD-WL and DSST per 10% increase of percentage energy from carbohydrates was (ORCERAD-WL 1.09 95% CI 1.04–1.13); (ORDSST 1.04 95% CI 1.00–1.09) in fully adjusted models.

**TABLE 2:** Association of dietary carbohydrates with poor cognitive performance among participants whose daily fasting duration was fewer than 16 h.

Cognitive test	Quartiles of percentage energy from carbohydrates				*P*_for trend_	Per 10% increase of percentage energy from carbohydrates	*P*-value
	Q1 (*N* = 523)	Q2 (*N* = 522)	Q3 (*N* = 522)	Q4 (*N* = 523)			
**CERAD-WL**							
Case/N	108/523	123/522	141/522	176/523			
Model 1 OR (95% CI)	1.00 (Reference)	1.08 (0.75–1.54)	1.18 (0.92–1.52)	2.37 (1.62–3.46)	<0.001	1.11 (1.07–1.16)	< 0.001
Model 2 OR (95% CI)	1.00 (Reference)	1.16 (0.79–1.69)	1.15 (0.83–1.59)	2.19 (1.35–3.56)	0.006	1.10 (1.04–1.16)	0.004
Model 3 OR (95% CI)	1.00 (Reference)	1.13 (0.76–1.68)	1.11 (0.79–1.54)	2.14 (1.29–3.55)	0.012	1.10 (1.04–1.16)	0.008
**CERAD-DR**							
Case/N	174/523	172/522	218/522	218/523			
Model 1 OR (95% CI)	1.00 (Reference)	0.99 (0.72–1.35)	1.21 (0.92–1.61)	1.66 (1.16–2.38)	0.004	1.06 (1.01–1.10)	0.025
Model 2 OR (95% CI)	1.00 (Reference)	1.01 (0.75–1.37)	1.20 (0.90–1.60)	1.51 (1.05–2.19)	0.028	1.04 (0.99–1.09)	0.139
Model 3 OR (95% CI)	1.00 (Reference)	1.00 (0.73–1.37)	1.20 (0.90–1.61)	1.51 (1.05–2.17)	0.030	1.04 (0.99–1.09)	0.137
**AF**							
Case/N	118/523	135/522	156/522	176/523			
Model 1 OR (95% CI)	1.00 (Reference)	0.90 (0.54–1.52)	1.14 (0.82–1.60)	1.38 (1.01–1.90)	0.011	1.04 (1.01–1.08)	0.005
Model 2 OR (95% CI)	1.00 (Reference)	0.98 (0.53–1.81)	1.13 (0.73–1.73)	1.20 (0.75–1.92)	0.331	1.03 (0.97–1.08)	0.323
Model 3 OR (95% CI)	1.00 (Reference)	0.96 (0.51–1.79)	1.10 (0.71–1.71)	1.18 (0.73–1.90)	0.395	1.02 (0.97–1.08)	0.377
**DSST**							
Case/N	101/523	112/522	147/522	168/523			
Model 1 OR(95% CI)	1.00 (Reference)	1.27 (0.85–1.91)	1.58 (1.13–2.22)	1.94 (1.39–2.71)	<0.001	1.09 (1.05–1.13)	<0.001
Model 2 OR (95% CI)	1.00 (Reference)	1.57 (1.01–2.46)	1.53 (1.01–2.31)	1.52 (1.05–2.19)	0.027	1.04 (1.00–1.09)	0.049
Model 3 OR(95% CI)	1.00 (Reference)	1.49 (0.97–2.30)	1.45 (0.95–2.24)	1.44 (1.00–2.11)	0.066	1.04 (1.00–1.09)	0.106

Model 1: adjusted for age, gender, ethnicity.

Model 2: additionally adjusted for income, education, exercise, current smoker, current drinker, supplement use, BMI, total intake of energy, AHEI, dietary fiber intake.

Model 3: additionally adjusted for self-reported of diabetes, hypertension, cardiovascular diseases (congestive heart failure, coronary heart disease, angina, heart attack, and stroke), cancer, and sleep disorders.

BMI, body mass index; AHEI, alternative healthy eating index; Q, quartile; CERAD-WL, Consortium to Establish a Registry for Alzheimer’s Disease Word List Learning Test; CERAD-DR, CERAD Word List Delayed Recall Test; AF, Animal Fluency; DSST, Digit Symbol Substitution Test.

The association of percentage energy from carbohydrates (%) with poor cognitive performance among participants whose daily fasting duration was more than 16 h is shown in Table 3. For the four cognitive tests, no association was observed for quartiles of percentage energy from carbohydrates and per 10% increase of percentage energy from carbohydrates and cognitive tests in all models.

**TABLE 3:** Association of dietary carbohydrates with poor cognitive performance among participants whose daily fasting duration was more than 16 h.

Cognitive test	Quartiles of percentage energy from carbohydrates				*P*_for trend_	Per 10% increase of percentage energy from carbohydrates	*P*-value
	Q1 (*N* = 99)	Q2 (*N* = 99)	Q3 (*N* = 99)	Q4 (*N* = 98)			
**CERAD-WL**							
Case/N	16/99	33/99	30/99	25/98			
Model 1 OR (95% CI)	1.00 (Reference)	3.18 (1.54–6.57)	2.01 (0.71–5.67)	1.35 (0.41–4.45)	0.819	1.02 (0.90–1.15)	0.733
Model 2 OR(95% CI)	1.00 (Reference)	4.06 (1.80–9.17)	1.94 (0.55–6.74)	1.25 (0.31–4.89)	0.994	1.02 (0.88–1.17)	0.798
Model 3 OR(95% CI)	1.00 (Reference)	3.85 (1.75–8.49)	1.87 (0.54–6.43)	1.20 (0.31–4.66)	0.960	1.01 (0.88–1.16)	0.873
**CERAD-DR**							
Case/N	22/99	38/99	30/99	34/98			
Model 1 OR (95% CI)	1.00 (Reference)	2.35 (1.25–4.43)	1.55 (0.85–2.84)	1.48 (0.63–3.42)	0.542	1.02 (0.93–1.12)	0.638
Model 2 OR(95% CI)	1.00 (Reference)	3.33 (1.64–6.75)	1.69 (0.76–3.77)	1.57 (0.70–3.48)	0.612	1.02 (0.92–1.12)	0.673
Model 3 OR (95% CI)	1.00 (Reference)	3.18 (1.70–5.93)	1.79 (0.82–3.88)	1.72 (0.76–3.88)	0.458	1.03 (0.93–1.14)	0.525
**AF**							
Case/N	18/99	37/99	27/99	30/98			
Model 1 OR(95% CI)	1.00 (Reference)	2.41 (1.06–5.46)	1.81 (0.79–4.14)	1.69 (0.79–3.62)	0.298	1.04 (0.95–1.13)	0.373
Model 2 OR (95% CI)	1.00 (Reference)	2.81 (1.33–5.90)	1.50 (0.62–3.63)	1.36 (0.73–2.55)	0.867	1.00 (0.90–1.12)	0.938
Model 3 OR (95% CI)	1.00 (Reference)	2.41 (1.11–5.18)	1.33 (0.56–3.19)	1.17 (0.62–2.20)	0.823	0.98 (0.88–1.09)	0.716
**DSST**							
Case/N	21/99	25/99	28/99	33/98			
Model 1 OR(95% CI)	1.00 (Reference)	0.92 (0.38–2.25)	1.36 (0.52–3.54)	1.72 (0.67–4.40)	0.226	1.08 (0.94–1.23)	0.256
Model 2 OR (95% CI)	1.00 (Reference)	0.98 (0.32–2.96)	1.04 (0.32–3.31)	1.24 (0.38–3.98)	0.731	1.03 (0.87–1.21)	0.724
Model 3 OR (95% CI)	1.00 (Reference)	0.74 (0.25–2.19)	0.95 (0.26–3.42)	1.02 (0.28–3.72)	0.885	1.01 (0.84–1.21)	0.886

Model 1: adjusted for age, gender, ethnicity.

Model 2: additionally adjusted for income, education, exercise, current smoker, current drinker, supplement use, BMI, total intake of energy, AHEI, dietary fiber intake.

Model 3: additionally adjusted for self-reported of diabetes, hypertension, cardiovascular diseases (congestive heart failure, coronary heart disease, angina, heart attack, and stroke), cancer, and sleep disorders.

BMI, body mass index; AHEI, alternative healthy eating index; Q, quartile; CERAD-WL, Consortium to Establish a Registry for Alzheimer’s Disease Word List Learning Test; CERAD-DR, CERAD Word List Delayed Recall Test; AF, Animal Fluency; DSST, Digit Symbol Substitution Test.

### The association of dietary carbohydrates with cognitive score among total participants, participants whose daily fasting duration was more or fewer than 16 h

Supplementary Table 2 shows the association of percentage energy from carbohydrates with cognitive scores among total participants. Compared with participants in quartile 1, participants in quartile 4 of percentage energy from carbohydrates were negatively associated with CERAD-WL score (βModel1 –1.23 95% CI –1.80; –0.65); (βModel2 –0.78 95% CI –1.46; –0.11); (βModel3 –0.75 95% CI –1.44; –0.06). Further, the highest quartile of percentage energy from carbohydrates was negatively associated with the CERAD-DR score (βModel1 –0.46 95% CI –0.76; –0.17) and AF score (βModel1 –1.32 95% CI –1.97; –0.67) when adjusted for age, gender, and ethnicity in Model 1. The association disappeared when additionally adjusted for other variables in Model 2 and Model 3. Moreover, participants in the highest quartile of percentage energy from carbohydrates was negatively associated with the DSST score (βModel3 –2.22 95% CI –4.41; –0.03) in the fully adjusted model. Additionally, when deciles of percentage energy from carbohydrates were present in a continuous form, a negative association was still observed between per 10% increase of percentage energy from carbohydrates and CERAD-WL score in the fully adjusted form (βModel3 –0.08 95% CI –0.16; –0.01).

The association of percentage energy from carbohydrates with cognitive score among participants whose daily fasting duration was fewer than 16 h is present in Supplementary Table 3. As shown in the table, participants in the highest quartile of percentage energy from carbohydrates were negatively associated with the CERAD-WL score (βModel1 –1.31 95% CI –2.09; –0.53); (βModel2 –0.92 95% CI –1.75; –0.09); (βModel3 –0.86 95% CI –1.70; –0.02) compared with the lowest quartile. After adjusting for age, gender, and ethnicity, there were negative associations between the highest quartile of percentage energy from carbohydrates and CERAD-DR score (βModel1 –0.45 95% CI –0.83; –0.07), AF score (βModel1 –1.36 95% CI –2.14; –0.57), and DSST score (βModel1 –5.38 95% CI –8.11; –2.65). No association was observed in Model 2 and Model 3. A negative association was observed between per 10% increase of percentage energy from carbohydrates and AF score in the fully adjusted form (βModel3 –0.10 95% CI –0.18; –0.02) when deciles of percentage energy from carbohydrates were shown in a continuous form.

The association of dietary carbohydrates consumption with cognitive scores among participants whose daily fasting duration was more than 16 h is shown in Supplementary Table 4. As shown in the table, no significant association was observed.

### Restricted cubic splines of percentage energy from carbohydrates (%) with poor cognitive performance among total participants, participants whose daily fasting duration was more or fewer than 16 h

**FIGURE 1:**
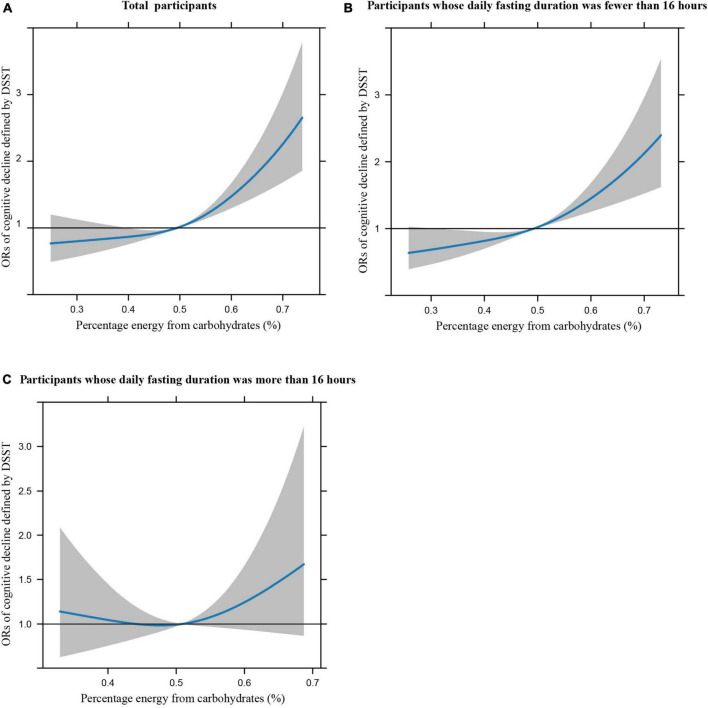
Restricted cubic splines between percentage energy from carbohydrates (%) and poor cognitive performance of CERAD-WL.

**FIGURE 2:**
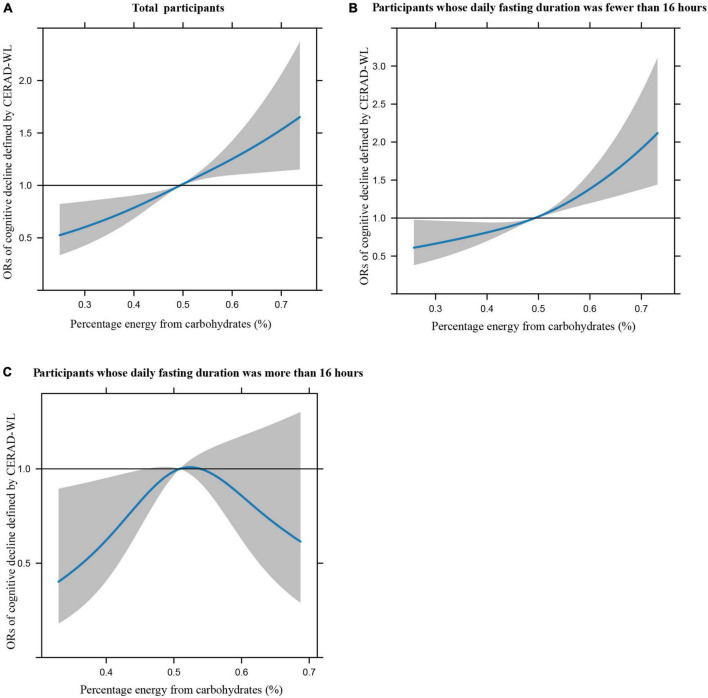
Restricted cubic splines between percentage energy from carbohydrates (%) and poor cognitive performance of CERAD-DR.

**FIGURE 3:**
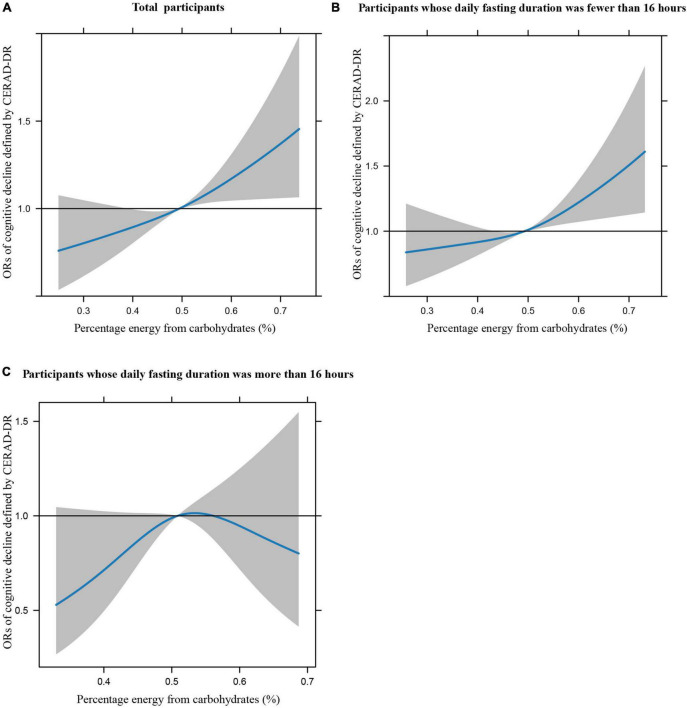
Restricted cubic splines between percentage energy from carbohydrates (%) and poor cognitive performance of AF.

**FIGURE 4:**
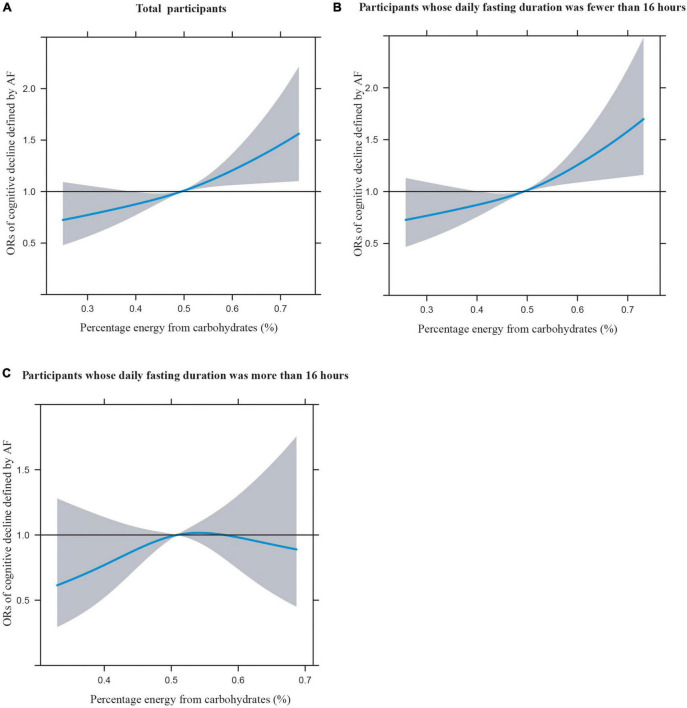
Restricted cubic splines between percentage energy from carbohydrates (%) and poor cognitive performance of DSST.

Figures 1A–C illustrates the restricted cubic splines of percentage energy from carbohydrates (%) with the cognitive decline of the CREAD-WL test among total participants, participants whose daily fasting duration was fewer or more than 16 h. There were significant positively linear dose-response associations between percentage energy from carbohydrates and cognitive decline of the CREAD-WL test among total participants (Figure 1A) (*P* non-linear = 0.696) and participants whose daily fasting duration was fewer than 16 h (Figure 1B) (*P* non-linear = 0.505), whereas a non-linear inverted “U” curve was observed among participants whose daily fasting duration was more than 16 h (Figure 1C) (*P* non-linear = 0.035). As shown in Figures 2A–C, 3A–C, similar dose-response associations were observed for the restricted cubic splines of the CERAD-DR and AF test among total participants (Figures 2A, 3A) (*P* non-linear for CERAD-DR = 0.721; *P* non-linear for AF = 0.702) and participants whose daily fasting duration was fewer than 16 h (Figures 2B, 3B) (*P* non-linear for CERAD-DR = 0.338; *P* non-linear for AF = 0.564), whereas no significant association was observed among participants whose daily fasting duration was more than 16 h (Figures 2C, 3C) (*P* non-linear for CERAD-DR = 0.134; *P* non-linear for AF = 0.319). For the cognitive decline of the DSST test in Figures 4A–C, a non-linear dose-response “L” curve was observed among total participants (Figure 4A) (*P* non-linear = 0.039) and a dose-response association was observed among participants whose daily fasting duration was fewer than 16 h (Figure 4B) (*P* non-linear = 0.276). Whereas, an “U” curve was observed among participants whose daily fasting duration was more than 16 h (Figure 4C) (*P* non-linear = 0.196).

### Sensitivity analyses

When excluding participants with sleep disorders, the association was consistent with the results of the total sample (Supplementary Tables 5–7). Meanwhile, the above association remained robust when additional adjustment was made for the timing of breakfast and dinner (Supplementary Tables 8–10). Further, the association between dietary carbohydrate consumption (g/day) with poor cognitive performance was consistent with the association for percentage energy from carbohydrates (Supplementary Tables 11–13). Finally, the association was remained robust between dietary carbohydrates and poor cognitive performance defined by total score of CERAD-WL, CERAD-DR, DSST, and AF test (Supplementary Tables 14–16). In addition, we found that consuming more carbohydrates were associated with cognitive performance among male, Non-Hispanic Black participants, and participants with or without regular exercise (Supplementary Tables 17–23).

## Discussion

This study investigates the association of dietary carbohydrates consumption with cognitive function among participants with different daily fasting duration among U.S older adults. In this nationally representative data, we found that higher percentage energy from carbohydrates was associated with poor cognitive performance, but not in participants whose daily fasting duration was more than 16 h. Further, the negative associations between dietary carbohydrates consumption and cognitive score were still significant among total participant and participants with a daily fasting duration of less than 16 h. Whereas, no association was observed among participants with a daily fasting duration of more than 16 h. In addition, dose-response associations were detected in the restricted cubic splines of percentage energy from carbohydrates and cognitive decline, while non-linear “U” curve associations were observed among participants whose daily fasting duration was more than 16 h.

To the best of our knowledge, this is the first study to illustrate that daily fasting duration could improve the cognitive impairment caused by dietary carbohydrates consumption. Previous studies have revealed that dietary carbohydrate consumption has been related to cognitive impairment, which was consistent with the current study among total participants (Bachlechner et al., 2017; Emilien et al., 2017; Hawkins et al., 2018). However, the long-term benefits and sustainability of the ketogenic diet have been controversial (O’Neill and Raggi, 2020). A growing body of evidence showed that time-restricted feeding (TRF; limiting the time of food consumption to 8–10 h throughout a day), a popular form of intermittent fasting, can improve cognitive function, aging, and extend healthy lifespan (Anton et al., 2019; Jamshed et al., 2019; Currenti et al., 2021; Ulgherait et al., 2021). Compared with the long-time diet control, there has been increased interest and acceptance of TRF, which may be easier to adhere to and maintain as time goes by Rynders et al. (2019). The direct effect of dietary carbohydrates and daily fasting duration on cognitive function has been widely documented respectively in previous studies (Benau et al., 2014; Hawkins et al., 2018; Muth and Park, 2021), whereas limited studies have assessed the association between dietary carbohydrates and cognitive function by considering daily fasting duration simultaneously. In the current study, we found that the association between dietary carbohydrates consumption and cognitive impairment could be improved by daily fasting duration among U.S older adults. When participants were stratified by daily fasting duration, the above association remained robust among participants with a daily fasting duration of fewer than 16 h, but not in participants with a daily fasting duration of more than 16 h. This observation illustrated that participants could attenuate the harmful effect of dietary carbohydrates on cognitive function by extending their daily fasting duration to 16 h. Similar associations were also observed when excluding participants with sleep disorders, which was a traditional risk factor for cognitive function (Irwin and Vitiello, 2019). Additionally, this study also observed that the percentage energy from carbohydrates was negatively associated with CERAD-WL and DSST scores among total participants and participants with a daily fasting duration of fewer than 16 h, but not in participants with a daily fasting duration of more than 16 h, which further supports our point of view.

The above association could be partially supported by the previous research. It appears that dietary carbohydrates on cognitive function include inflammatory, dysregulation in metabolic, and vascular factors (Hawkins et al., 2018). It has been one of the major public health concerns for establishing effective manners to prevent cognitive decline in older adults. To date, intermittent fasting has received increasing interest, which may produce biological changes similar to caloric restriction (Anton and Leeuwenburgh, 2013), as a useful manner to slow down the aging process and extend lifespan (Weindruch, 1996). Human studies showed the benefits of TRF meals on cognitive function and dementia in older adults (Anton et al., 2019; Balasubramanian et al., 2020; Currenti et al., 2021), which further supported the current study. Compared with traditional dietary restrictions, participants showed that this manner of eating was more acceptable and they would be willing to maintain this manner of eating pattern, suggesting intermittent fasting is a sustainable and useful strategy for older adults (Anton et al., 2019). A previous study suggested that intermittent fasting may counteract aging, which has been considered a major risk factor for cognitive impairment, dementia, and neurological disease (Longo and Mattson, 2014). Further, intermittent fasting has beneficial effects in increasing circulation, modulation of reactive oxygen species, and inflammation through increasing autophagy and mitochondrial respiratory efficiency (Lee and Longo, 2011). Animal studies also indicated that intermittent fasting was an effective mechanism for improving cognition impairment and hippocampal function in mice (Hamezah et al., 2019; Davis et al., 2021). A potential mechanism between intermittent fasting and cognitive function was that intermittent fasting was proved to reduce levels of inflammatory cytokines (TNFα, IL-1β, and IL-6), ROS production, and improve the function of endothelial (Arumugam et al., 2010; Headland et al., 2018). Moreover, intermittent fasting was reported to modulate the composition of microbial and increase its abundance, which had an influence on metabolism and nutritional status (Zeb et al., 2020). Therefore, the imbalance of the gut microbiome has been related to a series of immune, inflammatory, and nervous system-related diseases by the microbiome-brain axis (Salvucci, 2019), as well as the development and function of the brain (Ceppa et al., 2019).

This study also indicated the dose-response association between percentage energy from carbohydrates and cognitive impairment among total participants and participants whose daily fasting duration was fewer than 16 h by restricted cubic spline, while non-linear “U” curves were observed among participants whose daily fasting duration was more than 16 h, which may further support the findings in this study. In summary, this study illustrates that dietary carbohydrate consumption was associated with poor cognitive performance, while longer daily fasting duration could improve cognitive impairment. This study provides new perspectives and knowledge in the field of nutrition to prevent poor cognitive performance in the US elderly.

### Strengths and limitations

This study has several strengths. Firstly, this study firstly demonstrated the association between dietary carbohydrate consumption and cognitive impairment by simultaneously considering daily fasting duration based on the well-designed study (NHANES). Secondly, this study involved quite a large number of US older adults from the nationally representative data in the United States. Thirdly, a series of potential confounders were included in the current study to better examine the above association. Fourthly, this study also examined the dose-response association between percentage energy from carbohydrates and cognitive impairment among participants with different daily fasting duration. Finally, both quartiles and continuous forms of percentage energy from carbohydrates were considered in the current study. However, limitations still exist in this study. First of all, dietary information was adopted and measured by two 24-h dietary recalls, which cannot reflect the real consumption as usual. Secondly, causality could not be established due to the cross-sectional study design. Thirdly, residual confounders may still exist even if a large number of confounders were adjusted.

## Conclusion

This study indicated that dietary carbohydrates consumption was associated with poor cognitive performance, but not in participants whose daily fasting duration was more than 16 h among US older adults. The current analysis provides evidence that a longer daily fasting duration may improve the harmful effect of dietary carbohydrates on cognitive function.

## Data availability statement

Publicly available datasets were analyzed in this study. This data can be found here: https://wwwn.cdc.gov/nchs/nhanes/Default.aspx.

## Ethics statement

Ethical review and approval was not required for the study on human participants in accordance with the local legislation and institutional requirements. Written informed consent for participation was not required for this study in accordance with the national legislation and the institutional requirements.

## Author contributions

CS and WH designed the work and were responsible for the overall manuscript. SZ wrote the manuscript. TH conducted the statistical analysis. XP, YS, YZ, LL, and XW participated in data preparation. All authors made a significant contribution to this study, critically reviewed the manuscript, and approved the final published version.

## Abbreviations

AF: animal fluency
AHEI: Alternative Healthy Eating Index
BMI: Body Mass Index
CERAD-WL: Consortium to Establish a Registry for Alzheimer’s Disease Word List Learning
CERAD-DR: CERAD Word List Delayed Recall
CIs: confidence intervals
DSST: Digit Symbol Substitution Test
FNDDS: Food and Nutrient Database for Dietary Studies
MEC: Mobile Examination Center
NHANES: National Health and Nutrition Examination Survey
ORs: odds ratios.
