# Supplementary material for: The association of diet carbohydrates consumption with cognitive function among US older adults modification by daily fasting duration
Source: Front Aging Neurosci. 2022 Sep 26;14:991007. doi: 10.3389/fnagi.2022.991007 (PMC9550221; doi:10.3389/fnagi.2022.991007)
Supplement: Supplementary file 1 [file Data_Sheet_1.docx]

Supplementary Material

# Supplementary Figures and Tables

## Supplementary Tables

**Supplementary Table 1** **Characteristics of participants by the percentage of energy provided by carbohydrates (%).**

| Characteristics | Percentage of energy provided by carbohydrates (%) | | | | *P*-value |
| --- | --- | --- | --- | --- | --- |
|  | Q1 (N = 621) | Q2 (N = 621) | Q3 (N = 621) | Q4 (N = 622) |  |
| Age, years | 68.08 (67.56-68.60) | 69.04 (68.24-69.84) | 69.83 (69.03-70.62) | 70.08 (69.16-70.99) | 0.001 |
| Female, % | 50.05 (44.59-55.50) | 50.01 (44.38-55.62) | 59.80 (54.09-65.26) | 60.14 (54.72-65.32) | 0.122 |
| Non-Hispanic White, % | 85.54 (81.42-88.87) | 84.27 (80.52-87.41) | 80.51 (76.38-84.06) | 71.74 (64.94-77.67) | 0.083 |
| >$100000 annual household income, % | 26.96 (20.38-34.72) | 19.68 (13.90-27.12) | 20.69 (15.73-26.72) | 15.10 (10.28-21.64) | 0.036 |
| College graduate or above, % | 36.26 (28.91-44.31) | 31.61 (26.66-37.02) | 28.57 (23.19-34.64) | 25.73 (19.86-32.64) | 0.002 |
| Exercise regularly, % | 29.79 (24.09-36.19) | 23.34 (19.57-27.58) | 24.07 (19.20-29.73) | 23.48 (17.96-30.08) | 0.156 |
| Current smoker, % | 14.05 (11.03-17.75) | 11.29 (8.25-15.25) | 10.43 (7.84-13.74) | 9.43 (6.86-12.81) | 0.752 |
| Current drinker, % | 86.34 (82.81-89.24) | 77.30 (73.62-80.61) | 67.72 (61.85-73.09) | 55.80 (49.13-62.26) | <0.001 |
| BMI, kg/m^2^ | 28.96 (28.11-29.80) | 29.70 (28.86-30.54) | 29.25 (28.46-30.03) | 28.22 (27.66-28.77) | 0.007 |
| Dietary supplements use, % | 69.79 (63.99-75.02) | 69.94 (65.07-74.40) | 73.94 (68.37-78.83) | 71.96 (66.09-77.17) | 0.777 |
| Total energy, kcal/day | 1864.11 (1819.61-1908.62) | 2017.88 (1940.73-2095.03) | 1896.38 (1840.99-1951.77) | 1724.97 (1658.51-1791.43) | <0.001 |
| Fiber, g/day | 14.87 (14.24-15.50) | 17.71 (16.94-18.48) | 18.55 (17.68-19.41) | 19.32 (17.90-20.73) | <0.001 |
| AHEI | 52.17 (51.12-53.23) | 55.69 (54.31-57.06) | 52.82 (51.33-54.31) | 47.43 (45.97-48.89) | <0.001 |
| Diabetes, % | 19.26 (15.21-24.08) | 21.76 (17.80-26.32) | 17.16 (13.56-21.48) | 17.74 (14.58-21.42) | 0.139 |
| Hypertension, % | 58.95 (53.43-64.26) | 60.16 (55.66-64.50) | 54.52 (48.98-59.95) | 61.75 (56.51-66.73) | 0.005 |
| Cardiovascular diseases, % | 19.27 (15.67-23.48) | 22.69 (19.14-26.67) | 22.88 (19.80-26.29) | 21.88 (17.75-26.66) | 0.858 |
| Cancer, % | 26.04 (21.22-31.53) | 25.70 (20.69-31.44) | 23.60 (19.24-28.59) | 22.66 (18.10-27.97) | 0.435 |
| Sleep disorders, % | 12.70 (9.60-16.62) | 12.21 (9.40-15.72) | 12.35 (9.38-16.09) | 10.77 (8.37-13.75) | 0.473 |
| Poor cognitive performance defined by CERAD-WL, % | 16.32 (12.96-20.35) | 18.45 (14.75-22.83) | 20.30 (17.12-23.89) | 30.16 (24.99-35.88) | 0.003 |
| Poor cognitive performance defined by CERAD-DR, % | 29.60 (24.73-34.98) | 31.90 (25.38-39.21) | 34.42 (30.60-38.46) | 41.4 (35.67-47.38) | 0.100 |
| Poor cognitive performance defined by AF, % | 17.76 (14.33-21.81) | 18.05 (14.92-21.66) | 20.99 (17.63-24.78) | 26.89 (23.59-30.47) | <0.001 |
| Poor cognitive performance defined by DSST, % | 11.14 (8.29-14.82) | 11.51 (9.16-14.36) | 15.87 (13.16-19.02) | 20.53 (16.04-25.90) | 0.005 |

All data analyses in the present study were based on weighted estimates with sample weights provided by the NHANES. Continuous variables were presented as weighted means (95% CIs). Categorical variables were presented as weighted percentages (95% CIs). P values were calculated by general linear model for continuous variables adjusting for age and the chi-squared test for categorical variables.

BMI, body mass index; AHEI, Alternative Healthy Eating Index; CERAD-WL, Consortium to Establish a Registry for Alzheimer’s Disease Word List Learning Test; CERAD-DR, Consortium to Establish a Registry for Alzheimer’s Disease Word List Delayed Recall Test; AF, Animal Fluency test; DSST, Digit Symbol Substitution Test; Q, quartile.

**Supplementary Table 2 Association of dietary carbohydrates with cognitive score among total participants.**

| **Cognitive Test** | Quartiles of percentage energy from carbohydrates | | | | *P* _for trend_ | Per 10% increase of percentage energy from carbohydrates | *P* value |
| --- | --- | --- | --- | --- | --- | --- | --- |
|  | Q1 (N = 621) | Q2 (N = 621) | Q3 (N = 621) | Q4 (N = 622) |  |  |  |
| **CERAD-WL** |  |  |  |  |  |  |  |
| Model 1  β (95% CI) | 0.00 (Reference) | -0.17 (-0.70;0.35) | -0.33 (-0.77;0.10) | -1.23 (-1.80;-0.65) | <0.001 | -0.14 (-0.21;-0.08) | <0.001 |
| Model 2  β(95% CI) | 0.00 (Reference) | -0.25 (-0.78;0.28) | -0.25 (-0.71;0.20) | -0.78 (-1.46;-0.11) | 0.027 | -0.08 (-0.16;-0.01) | 0.037 |
| Model 3  β (95% CI) | 0.00 (Reference) | -0.22 (-0.77;0.32) | -0.22 (-0.67;0.22) | -0.75 (-1.44;-0.06) | 0.036 | -0.08 (-0.16;-0.01) | 0.045 |
| **CERAD-DR** |  |  |  |  |  |  |  |
| Model 1  β (95% CI) | 0.00 (Reference) | -0.05 (-0.35;0.24) | -0.15 (-0.40;0.10) | -0.46 (-0.76;-0.17) | 0.003 | -0.05 (-0.08;-0.01) | 0.012 |
| Model 2  β(95% CI) | 0.00 (Reference) | -0.08 (-0.36;0.19) | -0.11 (-0.34;0.12) | -0.25 (-0.56;0.04) | 0.097 | -0.02 (-0.06;0.02) | 0.298 |
| Model 3  β(95% CI) | 0.00 (Reference) | -0.06 (-0.34;0.21) | -0.11 (-0.34;0.12) | -0.23 (-0.55;0.07) | 0.130 | -0.02 (-0.06;0.02) | 0.334 |
| **AF** |  |  |  |  |  |  |  |
| Model 1  β(95% CI) | 0.00 (Reference) | -0.19 (-1.14;0.76) | -0.39 (-1.17;0.38) | -1.32 (-1.97;-0.67) | <0.001 | -0.15 (-0.23;-0.07) | 0.001 |
| Model 2  β (95% CI) | 0.00 (Reference) | -0.27 (-1.23;0.67) | -0.28 (-1.12;0.55) | -0.61 (-1.29;0.06) | 0.085 | -0.07 (-0.14;0.01) | 0.068 |
| Model 3  β (95% CI) | 0.00 (Reference) | -0.25 (-1.20;0.69) | -0.27 (-1.08;0.52) | -0.61 (-1.26;0.04) | 0.080 | -0.07 (-0.15;0.01) | 0.057 |
| **DSST** |  |  |  |  |  |  |  |
| Model 1  β(95% CI) | 0.00 (Reference) | -1.12 (-3.44;1.19) | -2.02 (-4.66;0.62) | -5.60 (-8.20;-3.00) | <0.001 | -0.65 (-1.01;-0.29) | 0.001 |
| Model 2  β(95% CI) | 0.00 (Reference) | -0.98 (-2.99;1.03) | -0.99 (-3.56;1.57) | -2.18 (-4.44;0.07) | 0.067 | -0.23 (-0.54;0.07) | 0.120 |
| Model 3  β (95% CI) | 0.00 (Reference) | -0.90 (-2.92;1.12) | -0.98 (-3.48;1.50) | -2.22 (-4.41;-0.03) | 0.056 | -0.24 (-0.53;0.05) | 0.102 |

Model 1: adjusted for age, gender, ethnicity

Model 2: additionally adjusted for income, education, exercise, current smoker, current drinker, supplement use, BMI, total intake of energy, AHEI, dietary fiber intake

Model 3: additionally adjusted for self-reported of diabetes, hypertension, cardiovascular diseases (congestive heart failure, coronary heart disease, angina, heart attack, and stroke), cancer, and sleep disorders.

BMI, body mass index; AHEI, alternative healthy eating index; Q, quartile; CERAD-WL, Consortium to Establish a Registry for Alzheimer’s Disease Word List Learning Test; CERAD-DR, CERAD Word List Delayed Recall Test; AF, Animal Fluency; DSST, Digit Symbol Substitution Test.

**Supplementary Table 3 Association of dietary carbohydrates with cognitive score among participants whose daily fasting duration was fewer than 16 hours.**

| **Cognitive Test** | Quartiles of percentage energy from carbohydrates | | | | *P* _for trend_ | Per 10% increase of percentage energy from carbohydrates | *P* value |
| --- | --- | --- | --- | --- | --- | --- | --- |
|  | Q1 (N = 523) | Q2 (N = 522) | Q3 (N = 522) | Q4 (N = 523) |  |  |  |
| **CERAD-WL** |  |  |  |  |  |  |  |
| Model 1  β (95% CI) | 0.00 (Reference) | -0.10 (-0.61;0.41) | -0.23 (-0.72;0.25) | -1.31 (-2.09;-0.53) | 0.001 | -0.15 (-0.23;-0.06) | 0.002 |
| Model 2  β (95% CI) | 0.00 (Reference) | -0.13 (-0.67;0.40) | -0.14 (-0.66;0.37) | -0.92 (-1.75;-0.09) | 0.035 | -0.09 (-0.19;-0.01) | 0.048 |
| Model 3  β(95% CI) | 0.00 (Reference) | -0.09 (-0.64;0.46) | -0.09 (-0.62;0.43) | -0.86 (-1.70;-0.02) | 0.053 | -0.09 (-0.18;0.01) | 0.063 |
| **CERAD-DR** |  |  |  |  |  |  |  |
| Model 1  β (95% CI) | 0.00 (Reference) | -0.01 (-0.28;0.26) | -0.15 (-0.42;0.11) | -0.45 (-0.83;-0.07) | 0.014 | -0.05 (-0.09;-0.01) | 0.042 |
| Model 2  β(95% CI) | 0.00 (Reference) | -0.02 (-0.28;0.25) | -0.10 (-0.36;0.16) | -0.25 (-0.61;0.12) | 0.158 | -0.02 (-0.07;0.03) | 0.379 |
| Model 3  β (95% CI) | 0.00 (Reference) | 0.01 (-0.26;0.28) | -0.09 (-0.36;0.18) | -0.21 (-0.60;0.16) | 0.219 | -0.02 (-0.06;0.03) | 0.437 |
| **AF** |  |  |  |  |  |  |  |
| Model 1  β(95% CI) | 0.00 (Reference) | -0.16 (-1.19;0.87) | -0.54 (-1.27;0.18) | -1.36 (-2.14;-0.57) | <0.001 | -0.17 (-0.25;-0.08) | <0.001 |
| Model 2  β(95% CI) | 0.00 (Reference) | -0.23 (-1.27;0.81) | -0.43 (-1.18;0.31) | -0.80 (-1.65;0.04) | 0.037 | -0.10 (-0.18;-0.02) | 0.022 |
| Model 3  β (95% CI) | 0.00 (Reference) | -0.19 (-1.22;0.83) | -0.40 (-1.11;0.30) | -0.77 (-1.59;0.04) | 0.039 | -0.10 (-0.18;-0.02) | 0.019 |
| **DSST** |  |  |  |  |  |  |  |
| Model 1  β (95% CI) | 0.00 (Reference) | -1.84 (-4.37;0.69) | -2.63 (-5.22;-0.05) | -5.38 (-8.11;-2.65) | <0.001 | -0.61 (-0.96;-0.28) | 0.001 |
| Model 2  β(95% CI) | 0.00 (Reference) | -1.61 (-4.04;0.81) | -1.57 (-4.17;1.02) | -2.40 (-4.92;0.12) | 0.055 | -0.25 (-0.55;0.05) | 0.094 |
| Model 3  β (95% CI) | 0.00 (Reference) | -1.42 (-3.83;0.98) | -1.46 (-4.00;1.07) | -2.32 (-4.79;0.14) | 0.058 | -0.24 (-0.54;0.05) | 0.098 |

Model 1: adjusted for age, gender, ethnicity

Model 2: additionally adjusted for income, education, exercise, current smoker, current drinker, supplement use, BMI, total intake of energy, AHEI, dietary fiber intake

Model 3: additionally adjusted for self-reported of diabetes, hypertension, cardiovascular diseases (congestive heart failure, coronary heart disease, angina, heart attack, and stroke), cancer, and sleep disorders.

BMI, body mass index; AHEI, alternative healthy eating index; Q, quartile; CERAD-WL, Consortium to Establish a Registry for Alzheimer’s Disease Word List Learning Test; CERAD-DR, CERAD Word List Delayed Recall Test; AF, Animal Fluency; DSST, Digit Symbol Substitution Test.

**Supplementary Table 4** **Association of dietary carbohydrates with cognitive score among participants whose daily fasting duration was more than 16 hours.**

| **Cognitive Test** | Quartiles of percentage energy from carbohydrates | | | | *P* _for trend_ | Per 10% increase of percentage energy from carbohydrates | *P* value |
| --- | --- | --- | --- | --- | --- | --- | --- |
|  | Q1 (N = 99) | Q2 (N = 99) | Q3 (N = 99) | Q4 (N = 98) |  |  |  |
| **CERAD-WL** |  |  |  |  |  |  |  |
| Model 1  β(95% CI) | 0.00 (Reference) | -1.83 (-3.70;0.03) | -1.01 (-2.80;0.77) | -1.01 (-3.49;1.46) | 0.499 | -0.11 (-0.39;0.17) | 0.430 |
| Model 2  β(95% CI) | 0.00 (Reference) | -2.26 (-3.90;-0.61) | -1.07 (-2.93;0.78) | -1.11 (-3.19;0.97) | 0.453 | -0.12 (-0.36;0.12) | 0.318 |
| Model 3  β(95% CI) | 0.00 (Reference) | -2.06 (-3.64;-0.48) | -0.97 (-2.88;0.92) | -0.95 (-3.01;1.10) | 0.550 | -0.09 (-0.32;0.14) | 0.417 |
| **CERAD-DR** |  |  |  |  |  |  |  |
| Model 1  β(95% CI) | 0.00 (Reference) | -0.79 (-1.62;0.03) | -0.37 (-1.12;0.38) | -0.49 (-1.62;0.63) | 0.512 | -0.05 (-0.18;0.08) | 0.448 |
| Model 2  β(95% CI) | 0.00 (Reference) | -1.08 (-1.82;-0.35) | -0.46 (-1.26;0.34) | -0.63 (-1.68;0.41) | 0.415 | -0.06 (-0.19;0.07) | 0.337 |
| Model 3  β(95% CI) | 0.00 (Reference) | -0.96 (-1.58;-0.34) | -0.46 (-1.20;0.27) | -0.61 (-1.52;0.30) | 0.371 | -0.05 (-0.17;0.06) | 0.322 |
| **AF** |  |  |  |  |  |  |  |
| Model 1  β(95% CI) | 0.00 (Reference) | -0.64 (-1.98;0.68) | -0.01 (-1.96;1.93) | 0.60 (-1.43;2.65) | 0.500 | 0.07 (-0.19;0.32) | 0.604 |
| Model 2  β(95% CI) | 0.00 (Reference) | -0.67 (-2.14;0.80) | 0.62 (-1.75;3.00) | 1.56 (-0.66;3.78) | 0.125 | 0.20 (-0.09;0.49) | 0.172 |
| Model 3  β(95% CI) | 0.00 (Reference) | -0.36 (-1.70;0.97) | 0.72 (-1.43;2.89) | 1.88 (-0.20;3.97) | 0.064 | 0.24 (-0.03;0.51) | 0.074 |
| **DSST** |  |  |  |  |  |  |  |
| Model 1  β(95% CI) | 0.00 (Reference) | -1.56 (-7.58;4.44) | -2.70 (-9.78;4.37) | -4.92 (-14.82;4.97) | 0.299 | -0.55 (-1.74;0.64) | 0.354 |
| Model 2  β(95% CI) | 0.00 (Reference) | -2.23 (-7.32;2.85) | -0.86 (-7.49;5.76) | -2.62 (-10.75;5.50) | 0.611 | -0.20 (-1.21;0.79) | 0.681 |
| Model 3  β(95% CI) | 0.00 (Reference) | -2.06 (-6.63;2.51) | -1.26 (-7.96;5.44) | -2.58 (-10.94;5.76) | 0.603 | -0.21 (-1.22;0.81) | 0.681 |

Model 1: adjusted for age, gender, ethnicity

Model 2: additionally adjusted for income, education, exercise, current smoker, current drinker, supplement use, BMI, total intake of energy, AHEI, dietary fiber intake

Model 3: additionally adjusted for self-reported of diabetes, hypertension, cardiovascular diseases (congestive heart failure, coronary heart disease, angina, heart attack, and stroke), cancer, and sleep disorders.

BMI, body mass index; AHEI, alternative healthy eating index; Q, quartile; CERAD-WL, Consortium to Establish a Registry for Alzheimer’s Disease Word List Learning Test; CERAD-DR, CERAD Word List Delayed Recall Test; AF, Animal Fluency; DSST, Digit Symbol Substitution Test.

**Supplementary Table 5** **Association of dietary carbohydrates with poor cognitive performance excluding participants with sleep disorder.**

| **Cognitive Test** | Quartiles of percentage energy from carbohydrates | | | | *P* _for trend_ | *P* _for interaction with meal-timing_ | Per 10% increase of percentage energy from carbohydrates | *P* value |
| --- | --- | --- | --- | --- | --- | --- | --- | --- |
|  | Q1 (N = 546) | Q2 (N = 545) | Q3 (N = 546) | Q4 (N = 546) |  |  |  |  |
| **CERAD-WL** |  |  |  |  |  |  |  |  |
| Case/N | 119/546 | 140/545 | 160/546 | 182/546 |  |  |  |  |
| Model 1  OR (95% CI) | 1.00 (Reference) | 1.17 (0.78-1.77) | 1.32 (1.08-1.61) | 2.27 (1.61-3.20) | <0.001 | 0.455 | 1.12 (1.07-1.16) | <0.001 |
| Model 2  OR (95% CI) | 1.00 (Reference) | 1.33 (0.86-2.04) | 1.30 (1.01-1.67) | 2.12 (1.40-3.23) | 0.002 | 0.767 | 1.09 (1.05-1.15) | 0.001 |
| Model 3  OR (95% CI) | 1.00 (Reference) | 1.29 (0.83-2.01) | 1.29 (1.01-1.65) | 2.08 (1.36-3.19) | 0.004 | 0.700 | 1.10 (1.05-1.15) | 0.002 |
| **CERAD-DR** |  |  |  |  |  |  |  |  |
| Case/N | 187/546 | 202/545 | 230/546 | 237/546 |  |  |  |  |
| Model 1  OR (95% CI) | 1.00 (Reference) | 1.07 (0.75-1.53) | 1.15 (0.87-1.51) | 1.62 (1.21-2.19) | 0.003 | 0.123 | 1.05 (1.01-1.09) | 0.027 |
| Model 2  OR (95% CI) | 1.00 (Reference) | 1.17 (0.82-1.66) | 1.16 (0.87-1.55) | 1.53 (1.11-2.12) | 0.023 | 0.247 | 1.03 (0.99-1.08) | 0.160 |
| Model 3  OR (95% CI) | 1.00 (Reference) | 1.16 (0.80-1.69) | 1.18 (0.88-1.58) | 1.53 (1.10-2.13) | 0.024 | 0.215 | 1.03 (0.99-1.08) | 0.154 |
| **AF** |  |  |  |  |  |  |  |  |
| Case/N | 128/546 | 154/545 | 167/546 | 196/546 |  |  |  |  |
| Model 1  OR (95% CI) | 1.00 (Reference) | 0.89 (0.56-1.42) | 1.04 (0.72-1.50) | 1.28 (0.93-1.77) | 0.070 | 0.005 | 1.03 (0.99-1.07) | 0.089 |
| Model 2  OR OR (95% CI) | 1.00 (Reference) | 1.03 (0.61-1.75) | 1.05 (0.68-1.60) | 1.13 (0.73-1.75) | 0.546 | 0.061 | 1.01 (0.96-1.06) | 0.724 |
| Model 3  OR (95% CI) | 1.00 (Reference) | 1.01 (0.59-1.73) | 1.05 (0.68-1.60) | 1.10 (0.70-1.73) | 0.634 | 0.045 | 1.01 (0.96-1.06) | 0.793 |
| **DSST** |  |  |  |  |  |  |  |  |
| Case/N | 113/546 | 118/545 | 149/546 | 180/546 |  |  |  |  |
| Model 1  OR (95% CI) | 1.00 (Reference) | 0.88 (0.61-1.27) | 1.28 (0.92-1.77) | 1.73 (1.15-2.61) | 0.005 | 0.011 | 1.09 (1.03-1.14) | 0.003 |
| Model 2  OR (95% CI) | 1.00 (Reference) | 1.09 (0.72-1.65) | 1.17 (0.83-1.65) | 1.25 (0.78-2.02) | 0.317 | 0.025 | 1.03 (0.98-1.08) | 0.318 |
| Model 3  OR (95% CI) | 1.00 (Reference) | 1.05 (0.68-1.61) | 1.17 (0.83-1.67) | 1.21 (0.74-1.97) | 0.385 | 0.028 | 1.02 (0.97-1.08) | 0.408 |

Model 1: adjusted for age, gender, ethnicity

Model 2: additionally adjusted for income, education, exercise, current smoker, current drinker, supplement use, BMI, total intake of energy, AHEI, dietary fiber intake

Model 3: additionally adjusted for self-reported of diabetes, hypertension, cardiovascular diseases (congestive heart failure, coronary heart disease, angina, heart attack, and stroke), and cancer.

BMI, body mass index; AHEI, alternative healthy eating index; Q, quartile; CERAD-WL, Consortium to Establish a Registry for Alzheimer’s Disease Word List Learning Test; CERAD-DR, CERAD Word List Delayed Recall Test; AF, Animal Fluency; DSST, Digit Symbol Substitution Test.

**Supplementary Table 6 Association of dietary carbohydrates with poor cognitive performance among participants whose daily fasting duration was fewer than 16 hours excluding participants with sleep disorder.**

| **Cognitive Test** | Quartiles of percentage energy from carbohydrates | | | | *P* _for trend_ | Per 10% increase of percentage energy from carbohydrates | *P* value |
| --- | --- | --- | --- | --- | --- | --- | --- |
|  | Q1 (N = 464) | Q2 (N = 457) | Q3 (N = 455) | Q4 (N = 469) |  |  |  |
| **CERAD-WL** |  |  |  |  |  |  |  |
| Case/N | 96/464 | 113/457 | 126/455 | 156/469 |  |  |  |
| Model 1  OR (95% CI) | 1.00 (Reference) | 1.19 (0.83-1.72) | 1.25 (0.95-1.65) | 2.53 (1.70-3.74) | <0.001 | 1.12 (1.07-1.17) | <0.001 |
| Model 2  OR (95% CI) | 1.00 (Reference) | 1.31 (0.89-1.94) | 1.26 (0.89-1.78) | 2.43 (1.46-4.05) | 0.004 | 1.11 (1.05-1.18) | 0.003 |
| Model 3  OR(95% CI) | 1.00 (Reference) | 1.27 (0.84-1.91) | 1.23 (0.87-1.76) | 2.35 (1.38-3.99) | 0.007 | 1.11 (1.04-1.18) | 0.005 |
| **CERAD-DR** |  |  |  |  |  |  |  |
| Case/N | 155/464 | 157/457 | 190/455 | 193/469 |  |  |  |
| Model 1  OR(95% CI) | 1.00 (Reference) | 1.05 (0.76-1.45) | 1.19 (0.88-1.62) | 1.66 (1.15-2.39) | 0.006 | 1.05 (1.01-1.10) | 0.038 |
| Model 2  OR (95% CI) | 1.00 (Reference) | 1.11 (0.80-1.54) | 1.22 (0.88-1.70) | 1.54 (1.05-2.26) | 0.032 | 1.04 (0.98-1.09) | 0.148 |
| Model 3  OR(95% CI) | 1.00 (Reference) | 1.10 (0.78-1.54) | 1.23 (0.88-1.72) | 1.53 (1.04-2.24) | 0.035 | 1.04 (0.98-1.09) | 0.152 |
| **AF** |  |  |  |  |  |  |  |
| Case/N | 106/464 | 123/457 | 132/455 | 162/469 |  |  |  |
| Model 1  OR(95% CI) | 1.00 (Reference) | 0.89 (0.52-1.53) | 1.05 (0.67-1.65) | 1.27 (0.88-1.83) | 0.121 | 1.03 (0.99-1.07) | 0.203 |
| Model 2  OR(95% CI) | 1.00 (Reference) | 1.02 (0.55-1.88) | 1.09 (0.66-1.82) | 1.18 (0.73-1.93) | 0.443 | 1.01 (0.96-1.07) | 0.621 |
| Model 3  OR(95% CI) | 1.00 (Reference) | 0.99 (0.54-1.83) | 1.08 (0.65-1.80) | 1.15 (0.70-1.89) | 0.527 | 1.01 (0.95-1.07) | 0.703 |
| **DSST** |  |  |  |  |  |  |  |
| Case/N | 92/464 | 102/457 | 129/455 | 153/469 |  |  |  |
| Model 1  OR(95% CI) | 1.00 (Reference) | 1.24 (0.82-1.87) | 1.47 (1.04-2.07) | 1.93 (1.33-2.80) | <0.001 | 1.08 (1.03-1.13) | 0.002 |
| Model 2  OR(95% CI) | 1.00 (Reference) | 1.63 (1.04-2.56) | 1.48 (0.98-2.25) | 1.63 (1.11-2.42) | 0.016 | 1.04 (0.99-1.09) | 0.075 |
| Model 3  OR(95% CI) | 1.00 (Reference) | 1.55 (1.01-2.39) | 1.45 (0.94-2.22) | 1.55 (1.04-2.32) | 0.037 | 1.04 (0.99-1.08) | 0.152 |

Model 1: adjusted for age, gender, ethnicity

Model 2: additionally adjusted for income, education, exercise, current smoker, current drinker, supplement use, BMI, total intake of energy, AHEI, dietary fiber intake

Model 3: additionally adjusted for self-reported of diabetes, hypertension, cardiovascular diseases (congestive heart failure, coronary heart disease, angina, heart attack, and stroke), and cancer.

BMI, body mass index; AHEI, alternative healthy eating index; Q, quartile; CERAD-WL, Consortium to Establish a Registry for Alzheimer’s Disease Word List Learning Test; CERAD-DR, CERAD Word List Delayed Recall Test; AF, Animal Fluency; DSST, Digit Symbol Substitution Test.

**Supplementary Table 7 Association of dietary carbohydrates with poor cognitive performance among participants whose daily fasting duration was more than 16 hours excluding participants with sleep disorder.**

| **Cognitive Test** | Quartiles of percentage energy from carbohydrates | | | | *P* _for trend_ | Per 10% increase of percentage energy from carbohydrates | *P* value |
| --- | --- | --- | --- | --- | --- | --- | --- |
|  | Q1 (N = 82) | Q2 (N = 83) | Q3 (N = 84) | Q4 (N = 88) |  |  |  |
| **CERAD-WL** |  |  |  |  |  |  |  |
| Case/N | 12/82 | 28/83 | 28/84 | 21/88 |  |  |  |
| Model 1  OR (95CI%) | 1.00 (Reference) | 4.93 (2.14-11.33) | 2.94 (1.14-7.58) | 1.61 (0.56-4.58) | 0.717 | 1.04 (0.92-1.16) | 0.541 |
| Model 2  OR (95% CI) | 1.00 (Reference) | 5.94 (2.44-14.43) | 2.66 (0.85-8.25) | 1.38 (0.38-4.97) | 0.981 | 1.03 (0.90-1.18) | 0.669 |
| Model 3  OR(95% CI) | 1.00 (Reference) | 5.48 (2.31-12.98) | 2.68 (0.91-7.92) | 1.36 (0.39-4.67) | 0.983 | 1.03 (0.89-1.17) | 0.723 |
| **CERAD-DR** |  |  |  |  |  |  |  |
| Case/N | 20/82 | 31/83 | 25/84 | 31/88 |  |  |  |
| Model 1  OR(95% CI) | 1.00 (Reference) | 2.14 (0.80-5.72) | 1.13 (0.50-2.52) | 1.41 (0.58-3.39) | 0.793 | 1.02 (0.93-1.12) | 0.720 |
| Model 2  OR(95% CI) | 1.00 (Reference) | 2.73 (1.05-7.11) | 1.16 (0.42-3.20) | 1.52 (0.53-4.37) | 0.822 | 1.03 (0.91-1.16) | 0.677 |
| Model 3  OR(95% CI) | 1.00 (Reference) | 2.37 (1.01-5.53) | 1.23 (0.41-3.64) | 1.55 (0.51-4.70) | 0.737 | 1.03 (0.89-1.18) | 0.701 |
| **AF** |  |  |  |  |  |  |  |
| Case/N | 14/82 | 28/83 | 24/84 | 27/88 |  |  |  |
| Model 1  OR(95% CI) | 1.00 (Reference) | 2.24 (0.85-5.90) | 1.86 (0.76-4.56) | 1.56 (0.70-3.49) | 0.347 | 1.03 (0.95-1.13) | 0.444 |
| Model 2  OR (95% CI) | 1.00 (Reference) | 2.43 (1.02-5.79) | 1.43 (0.53-3.86) | 1.13 (0.61-2.09) | 0.822 | 0.99 (0.89-1.09) | 0.778 |
| Model 3  OR(95% CI) | 1.00 (Reference) | 2.10 (0.87-5.06) | 1.32 (0.48-3.62) | 1.03 (0.56-1.91) | 0.674 | 0.97 (0.88-1.07) | 0.543 |
| **DSST** |  |  |  |  |  |  |  |
| Case/N | 18/82 | 18/83 | 26/84 | 29/88 |  |  |  |
| Model 1  OR(95% CI) | 1.00 (Reference) | 0.78 (0.27-2.25) | 1.37 (0.52-3.63) | 1.57 (0.57-4.30) | 0.297 | 1.08 (0.94-1.24) | 0.289 |
| Model 2  OR (95% CI) | 1.00 (Reference) | 0.83 (0.23-2.95) | 1.06 (0.35-3.25) | 1.05 (0.34-3.23) | 0.839 | 1.02 (0.87-1.19) | 0.830 |
| Model 3  OR(95% CI) | 1.00 (Reference) | 0.58 (0.17-2.01) | 0.99 (0.29-3.35) | 0.80 (0.23-2.78) | 0.960 | 0.99 (0.84-1.18) | 0.972 |

Model 1: adjusted for age, gender, ethnicity

Model 2: additionally adjusted for income, education, exercise, current smoker, current drinker, supplement use, BMI, total intake of energy, AHEI, dietary fiber intake

Model 3: additionally adjusted for self-reported of diabetes, hypertension, cardiovascular diseases (congestive heart failure, coronary heart disease, angina, heart attack, and stroke), and cancer.

BMI, body mass index; AHEI, alternative healthy eating index; Q, quartile; CERAD-WL, Consortium to Establish a Registry for Alzheimer’s Disease Word List Learning Test; CERAD-DR, CERAD Word List Delayed Recall Test; AF, Animal Fluency; DSST, Digit Symbol Substitution Test.

**Supplementary Table 8** **Association of dietary carbohydrates with poor cognitive performance among total participants additionally adjusted with timing for breakfast and dinner.**

| **Cognitive Test** | Quartiles of percentage energy from carbohydrates | | | | *P* _for trend_ | *P* _for interaction with meal-timing_ | Per 10% increase of percentage energy from carbohydrates | *P* value |
| --- | --- | --- | --- | --- | --- | --- | --- | --- |
|  | Q1 (N = 621) | Q2 (N = 621) | Q3 (N = 621) | Q4 (N = 621) |  |  |  |  |
| **CERAD-WL** |  |  |  |  |  |  |  |  |
| Case/N | 135/621 | 154/621 | 177/621 | 209/621 |  |  |  |  |
| Model 1  OR (95% CI) | 1.00 (Reference) | 1.07 (0.73-1.57) | 1.20 (0.99-1.47) | 2.09 (1.55-2.81) | <0.001 | 0.457 | 1.10 (1.06-1.14) | <0.001 |
| Model 2  OR (95% CI) | 1.00 (Reference) | 1.18 (0.80-1.74) | 1.18 (0.93-1.50) | 1.87 (1.28-2.73) | 0.004 | 0.886 | 1.08 (1.04-1.13) | 0.002 |
| Model 3  OR (95% CI) | 1.00 (Reference) | 1.14 (0.76-1.72) | 1.15 (0.91-1.45) | 1.79 (1.22-2.63) | 0.010 | 0.796 | 1.08 (1.04-1.13) | 0.004 |
| **CERAD-DR** |  |  |  |  |  |  |  |  |
| Case/N | 213/621 | 224/621 | 264/621 | 272/621 |  |  |  |  |
| Model 1  OR (95% CI) | 1.00 (Reference) | 1.04 (0.75-1.44) | 1.18 (0.90-1.55) | 1.60 (1.23-2.08) | 0.002 | 0.185 | 1.05 (1.02-1.09) | 0.009 |
| Model 2  OR (95% CI) | 1.00 (Reference) | 1.09 (0.80-1.50) | 1.17 (0.90-1.53) | 1.44 (1.10-1.90) | 0.018 | 0.370 | 1.03 (0.99-1.07) | 0.101 |
| Model 3  OR (95% CI) | 1.00 (Reference) | 1.07 (0.77-1.49) | 1.17 (0.89-1.55) | 1.38 (1.06-1.81) | 0.033 | 0.332 | 1.03 (0.99-1.07) | 0.152 |
| **AF** |  |  |  |  |  |  |  |  |
| Case/N | 145/621 | 175/621 | 190/621 | 219/621 |  |  |  |  |
| Model 1  OR (95% CI) | 1.00 (Reference) | 0.95 (0.62-1.45) | 1.07 (0.81-1.42) | 1.44 (1.09-1.90) | 0.003 | 0.006 | 1.05 (1.02-1.07) | 0.001 |
| Model 2  OR (95% CI) | 1.00 (Reference) | 1.05 (0.63-1.75) | 1.06 (0.73-1.53) | 1.20 (0.80-1.82) | 0.339 | 0.069 | 1.02 (0.98-1.06) | 0.382 |
| Model 3  OR (95% CI) | 1.00 (Reference) | 1.03 (0.61-1.72) | 1.05 (0.72-1.51) | 1.15 (0.74-1.78) | 0.490 | 0.048 | 1.01 (0.97-1.06) | 0.540 |
| **DSST** |  |  |  |  |  |  |  |  |
| Case/N | 123/621 | 131/621 | 165/621 | 204/621 |  |  |  |  |
| Model 1  OR (95% CI) | 1.00 (Reference) | 0.94 (0.69-1.30) | 1.33 (0.97-1.84) | 1.82 (1.28-2.59) | <0.001 | 0.010 | 1.09 (1.05-1.14) | <0.001 |
| Model 2  OR (95% CI) | 1.00 (Reference) | 1.12 (0.79-1.59) | 1.24 (0.88-1.76) | 1.26 (0.86-1.85) | 0.197 | 0.055 | 1.03 (0.99-1.08) | 0.148 |
| Model 3  OR (95% CI) | 1.00 (Reference) | 1.08 (0.75-1.55) | 1.20 (0.84-1.71) | 1.22 (0.80-1.86) | 0.310 | 0.038 | 1.03 (0.98-1.08) | 0.248 |

Model 1: adjusted for age, gender, ethnicity

Model 2: additionally adjusted for income, education, exercise, current smoker, current drinker, supplement use, BMI, total intake of energy, AHEI, dietary fiber intake

Model 3: additionally adjusted for self-reported of diabetes, hypertension, cardiovascular diseases (congestive heart failure, coronary heart disease, angina, heart attack, and stroke), cancer, sleep disorders, timing for breakfast, and timing for dinner.

BMI, body mass index; AHEI, alternative healthy eating index; Q, quartile; CERAD-WL, Consortium to Establish a Registry for Alzheimer’s Disease Word List Learning Test; CERAD-DR, CERAD Word List Delayed Recall Test; AF, Animal Fluency; DSST, Digit Symbol Substitution Test.

**Supplementary Table 9 Association of dietary carbohydrates with poor cognitive performance among participants whose daily fasting duration was fewer than 16 hours additionally adjusted with timing for breakfast and dinner.**

| **Cognitive Test** | Quartiles of percentage energy from carbohydrates | | | | *P* _for trend_ | Per 10% increase of percentage energy from carbohydrates | *P* value |
| --- | --- | --- | --- | --- | --- | --- | --- |
|  | Q1 (N = 523) | Q2 (N = 522) | Q3 (N = 522) | Q4 (N = 523) |  |  |  |
| **CERAD-WL** |  |  |  |  |  |  |  |
| Case/N | 108/523 | 123/522 | 141/522 | 176/523 |  |  |  |
| Model 1  OR (95% CI) | 1.00 (Reference) | 1.08 (0.75-1.54) | 1.18 (0.92-1.52) | 2.37 (1.62-3.46) | <0.001 | 1.11 (1.07-1.16) | <0.001 |
| Model 2  OR (95% CI) | 1.00 (Reference) | 1.16 (0.79-1.69) | 1.15 (0.83-1.59) | 2.19 (1.35-3.56) | 0.006 | 1.10 (1.04-1.16) | 0.004 |
| Model 3  OR (95% CI) | 1.00 (Reference) | 1.13 (0.76-1.69) | 1.11 (0.79-1.54) | 2.13 (1.29-3.50) | 0.013 | 1.09 (1.03-1.16) | 0.009 |
| **CERAD-DR** |  |  |  |  |  |  |  |
| Case/N | 174/523 | 172/522 | 218/522 | 218/523 |  |  |  |
| Model 1  OR (95% CI) | 1.00 (Reference) | 0.99 (0.72-1.35) | 1.21 (0.92-1.61) | 1.66 (1.16-2.38) | 0.004 | 1.06 (1.01-1.10) | 0.025 |
| Model 2  OR (95% CI) | 1.00 (Reference) | 1.01 (0.75-1.37) | 1.20 (0.90-1.60) | 1.51 (1.05-2.19) | 0.028 | 1.04 (0.99-1.09) | 0.139 |
| Model 3  OR (95% CI) | 1.00 (Reference) | 0.98 (0.72-1.34) | 1.19 (0.88-1.61) | 1.43 (1.01-2.04) | 0.046 | 1.03 (0.99-1.08) | 0.191 |
| **AF** |  |  |  |  |  |  |  |
| Case/N | 118/523 | 135/522 | 156/522 | 176/523 |  |  |  |
| Model 1  OR (95% CI) | 1.00 (Reference) | 0.90 (0.54-1.52) | 1.14 (0.82-1.60) | 1.38 (1.01-1.90) | 0.011 | 1.04 (1.01-1.08) | 0.005 |
| Model 2  OR (95% CI) | 1.00 (Reference) | 0.98 (0.53-1.81) | 1.13 (0.73-1.73) | 1.20 (0.75-1.92) | 0.331 | 1.03 (0.97-1.08) | 0.323 |
| Model 3  OR (95% CI) | 1.00 (Reference) | 0.95 (0.51-1.78) | 1.11 (0.71-1.71) | 1.14 (0.69-1.86) | 0.487 | 1.02 (0.97-1.07) | 0.473 |
| **DSST** |  |  |  |  |  |  |  |
| Case/N | 101/523 | 112/522 | 147/522 | 168/523 |  |  |  |
| Model 1  OR (95% CI) | 1.00 (Reference) | 1.27 (0.85-1.91) | 1.58 (1.13-2.22) | 1.94 (1.39-2.71) | <0.001 | 1.09 (1.05-1.13) | <0.001 |
| Model 2  OR (95% CI) | 1.00 (Reference) | 1.57 (1.01-2.46) | 1.53 (1.01-2.31) | 1.52 (1.05-2.19) | 0.027 | 1.04 (1.00-1.09) | 0.049 |
| Model 3  OR (95% CI) | 1.00 (Reference) | 1.51 (0.96-2.34) | 1.45 (0.94-2.22) | 1.47 (1.01-2.16) | 0.059 | 1.04 (1.00-1.09) | 0.097 |

Model 1: adjusted for age, gender, ethnicity

Model 2: additionally adjusted for income, education, exercise, current smoker, current drinker, supplement use, BMI, total intake of energy, AHEI, dietary fiber intake

Model 3: additionally adjusted for self-reported of diabetes, hypertension, cardiovascular diseases (congestive heart failure, coronary heart disease, angina, heart attack, and stroke), cancer, sleep disorders, timing for breakfast, and timing for dinner.

BMI, body mass index; AHEI, alternative healthy eating index; Q, quartile; CERAD-WL, Consortium to Establish a Registry for Alzheimer’s Disease Word List Learning Test; CERAD-DR, CERAD Word List Delayed Recall Test; AF, Animal Fluency; DSST, Digit Symbol Substitution Test.

**Supplementary Table 10 Association of dietary carbohydrates with poor cognitive performance among participants whose daily fasting duration was more than 16 hours additionally adjusted with timing for breakfast and dinner.**

| **Cognitive Test** | Quartiles of percentage energy from carbohydrates | | | | *P* _for trend_ | Per 10% increase of percentage energy from carbohydrates | *P* value |
| --- | --- | --- | --- | --- | --- | --- | --- |
|  | Q1 (N = 99) | Q2 (N = 99) | Q3 (N = 99) | Q4 (N = 98) |  |  |  |
| **CERAD-WL** |  |  |  |  |  |  |  |
| Case/N | 16/99 | 33/99 | 30/99 | 25/98 |  |  |  |
| Model 1  OR (95% CI) | 1.00 (Reference) | 3.18 (1.54-6.57) | 2.01 (0.71-5.67) | 1.35 (0.41-4.45) | 0.819 | 1.02 (0.90-1.15) | 0.733 |
| Model 2  OR (95% CI) | 1.00 (Reference) | 4.06 (1.80-9.17) | 1.94 (0.55-6.74) | 1.25 (0.31-4.89) | 0.994 | 1.02 (0.88-1.17) | 0.798 |
| Model 3  OR (95% CI) | 1.00 (Reference) | 3.96 (1.80-8.69) | 1.92 (0.55-6.67) | 1.22 (0.31-4.77) | 0.980 | 1.01 (0.88-1.16) | 0.866 |
| **CERAD-DR** |  |  |  |  |  |  |  |
| Case/N | 22/99 | 38/99 | 30/99 | 34/98 |  |  |  |
| Model 1  OR (95% CI) | 1.00 (Reference) | 2.35 (1.25-4.43) | 1.55 (0.85-2.84) | 1.48 (0.63-3.42) | 0.542 | 1.02 (0.93-1.12) | 0.638 |
| Model 2  OR (95% CI) | 1.00 (Reference) | 3.33 (1.64-6.75) | 1.69 (0.76-3.77) | 1.57 (0.70-3.48) | 0.612 | 1.02 (0.92-1.12) | 0.673 |
| Model 3  OR (95% CI) | 1.00 (Reference) | 3.24 (1.71-6.14) | 1.83 (0.85-3.91) | 1.75 (0.78-3.89) | 0.428 | 1.04 (0.94-1.14) | 0.504 |
| **AF** |  |  |  |  |  |  |  |
| Case/N | 18/99 | 37/99 | 27/99 | 30/98 |  |  |  |
| Model 1  OR (95% CI) | 1.00 (Reference) | 2.41 (1.06-5.46) | 1.81 (0.79-4.14) | 1.69 (0.79-3.62) | 0.298 | 1.04 (0.95-1.13) | 0.373 |
| Model 2  OR (95% CI) | 1.00 (Reference) | 2.81 (1.33-5.90) | 1.50 (0.62-3.63) | 1.36 (0.73-2.55) | 0.867 | 1.00 (0.90-1.12) | 0.938 |
| Model 3  OR (95% CI) | 1.00 (Reference) | 2.32 (1.10-4.90) | 1.31 (0.57-3.02) | 1.13 (0.61-2.09) | 0.756 | 0.98 (0.89-1.08) | 0.675 |
| **DSST** |  |  |  |  |  |  |  |
| Case/N | 21/99 | 25/99 | 28/99 | 33/98 |  |  |  |
| Model 1  OR (95% CI) | 1.00 (Reference) | 0.92 (0.38-2.25) | 1.36 (0.52-3.54) | 1.72 (0.67-4.40) | 0.226 | 1.08 (0.94-1.23) | 0.256 |
| Model 2  OR (95% CI) | 1.00 (Reference) | 0.98 (0.32-2.96) | 1.04 (0.32-3.31) | 1.24 (0.38-3.98) | 0.731 | 1.03 (0.87-1.21) | 0.724 |
| Model 3  OR (95% CI) | 1.00 (Reference) | 0.73 (0.23-2.26) | 0.96 (0.24-3.77) | 1.01 (0.25-3.86) | 0.909 | 1.01 (0.83-1.22) | 0.918 |

Model 1: adjusted for age, gender, ethnicity

Model 2: additionally adjusted for income, education, exercise, current smoker, current drinker, supplement use, BMI, total intake of energy, AHEI, dietary fiber intake

Model 3: additionally adjusted for self-reported of diabetes, hypertension, cardiovascular diseases (congestive heart failure, coronary heart disease, angina, heart attack, and stroke), cancer, sleep disorders, timing for breakfast, and timing for dinner.

BMI, body mass index; AHEI, alternative healthy eating index; Q, quartile; CERAD-WL, Consortium to Establish a Registry for Alzheimer’s Disease Word List Learning Test; CERAD-DR, CERAD Word List Delayed Recall Test; AF, Animal Fluency; DSST, Digit Symbol Substitution Test.

**Supplementary Table 11 Association of dietary carbohydrates with poor cognitive performance among total participants.**

| **Cognitive Test** | Quartiles of carbohydrates consumption | | | | *P* _for trend_ | *P* _for interaction with meal-timing_ | Per 10% increase of carbohydrates consumption | *P* value |
| --- | --- | --- | --- | --- | --- | --- | --- | --- |
|  | Q1 (N = 622) | Q2 (N = 621) | Q3 (N = 621) | Q4 (N = 621) |  |  |  |  |
| **CERAD-WL** |  |  |  |  |  |  |  |  |
| Case/N | 178/622 | 161/621 | 164/621 | 172/621 |  |  |  |  |
| Model 1  OR (95% CI) | 1.00 (Reference) | 0.86 (0.62-1.20) | 0.70 (0.50-1.00) | 0.83 (0.61-1.12) | 0.197 | 0.099 | 0.97 (0.93-1.01) | 0.094 |
| Model 2  OR (95% CI) | 1.00 (Reference) | 1.17 (0.78-1.75) | 1.32 (0.82-2.13) | 2.31 (1.36-3.94) | 0.003 | 0.205 | 1.12 (1.05-1.19) | 0.002 |
| Model 3  OR (95% CI) | 1.00 (Reference) | 1.17 (0.79-1.74) | 1.33 (0.81-2.17) | 2.25 (1.32-3.83) | 0.005 | 0.210 | 1.12 (1.05-1.19) | 0.004 |
| **CERAD-DR** |  |  |  |  |  |  |  |  |
| Case/N | 261/622 | 224/621 | 240/621 | 248/621 |  |  |  |  |
| Model 1  OR (95% CI) | 1.00 (Reference) | 0.73 (0.52-1.04) | 0.75 (0.57-1.01) | 0.85 (0.60-1.23) | 0.524 | 0.021 | 0.98 (0.93-1.02) | 0.289 |
| Model 2  OR (95% CI) | 1.00 (Reference) | 0.90 (0.64-1.27) | 1.16 (0.84-1.60) | 1.69 (1.07-2.65) | 0.020 | 0.065 | 1.07 (1.01-1.14) | 0.057 |
| Model 3  OR (95% CI) | 1.00 (Reference) | 0.92 (0.66-1.30) | 1.20 (0.87-1.66) | 1.74 (1.12-2.71) | 0.018 | 0.073 | 1.07 (1.01-1.14) | 0.053 |
| **AF** |  |  |  |  |  |  |  |  |
| Case/N | 219/622 | 200/621 | 185/621 | 125/621 |  |  |  |  |
| Model 1  OR (95% CI) | 1.00 (Reference) | 0.78 (0.60-1.03) | 0.68 (0.54-0.86) | 0.50 (0.35-0.70) | <0.001 | 0.158 | 0.92 (0.88-0.96) | <0.001 |
| Model 2  OR (95% CI) | 1.00 (Reference) | 0.98 (0.70-1.38) | 1.16 (0.77-1.72) | 1.16 (0.64-2.10) | 0.572 | 0.464 | 1.06 (0.97-1.16) | 0.208 |
| Model 3  OR (95% CI) | 1.00 (Reference) | 0.99 (0.70-1.41) | 1.17 (0.77-1.76) | 1.14 (0.61-2.11) | 0.651 | 0.508 | 1.06 (0.97-1.16) | 0.241 |
| **DSST** |  |  |  |  |  |  |  |  |
| Case/N | 199/622 | 165/621 | 155/621 | 104/621 |  |  |  |  |
| Model 1  OR (95% CI) | 1.00 (Reference) | 0.62 (0.45-0.85) | 0.52 (0.38-0.71) | 0.28 (0.18-0.44) | <0.001 | 0.475 | 0.86 (0.82-0.90) | <0.001 |
| Model 2  OR (95% CI) | 1.00 (Reference) | 0.73 (0.48-1.09) | 0.89 (0.49-1.61) | 0.72 (0.32-1.62) | 0.520 | 0.965 | 0.99 (0.91-1.09) | 0.970 |
| Model 3  OR (95% CI) | 1.00 (Reference) | 0.73 (0.49-1.09) | 0.89 (0.48-1.63) | 0.68 (0.30-1.54) | 0.439 | 0.978 | 0.99 (0.91-1.09) | 0.937 |

Model 1: adjusted for age, gender, ethnicity

Model 2: additionally adjusted for income, education, exercise, current smoker, current drinker, supplement use, BMI, total intake of energy, AHEI, dietary fiber intake

Model 3: additionally adjusted for self-reported of diabetes, hypertension, cardiovascular diseases (congestive heart failure, coronary heart disease, angina, heart attack, and stroke), cancer, and sleep disorders.

BMI, body mass index; AHEI, alternative healthy eating index; Q, quartile; CERAD-WL, Consortium to Establish a Registry for Alzheimer’s Disease Word List Learning Test; CERAD-DR, CERAD Word List Delayed Recall Test; AF, Animal Fluency; DSST, Digit Symbol Substitution Test.

**Supplementary Table 12 Association of dietary carbohydrates with poor cognitive performance among participants whose daily fasting duration was fewer than 16 hours.**

| **Cognitive Test** | Quartiles of percentage energy from carbohydrates | | | | *P* _for trend_ | Per 10% increase of percentage energy from carbohydrates | *P* value |
| --- | --- | --- | --- | --- | --- | --- | --- |
|  | Q1 (N = 522) | Q2 (N = 523) | Q3 (N = 523) | Q4 (N = 522) |  |  |  |
| **CERAD-WL** |  |  |  |  |  |  |  |
| Case/N | 144/522 | 129/523 | 132/523 | 143/522 |  |  |  |
| Model 1  OR (95% CI) | 1.00 (Reference) | 0.82 (0.57-1.18) | 0.65 (0.45-0.93) | 0.85 (0.60-1.20) | 0.325 | 0.96 (0.92-1.01) | 0.122 |
| Model 2  OR (95% CI) | 1.00 (Reference) | 1.12 (0.73-1.72) | 1.22 (0.75-1.97) | 2.31 (1.33-4.04) | 0.005 | 1.09 (1.01-1.17) | 0.052 |
| Model 3  OR (95% CI) | 1.00 (Reference) | 1.12 (0.73-1.71) | 1.21 (0.74-1.99) | 2.20 (1.25-3.85) | 0.012 | 1.08 (1.00-1.17) | 0.081 |
| **CERAD-DR** |  |  |  |  |  |  |  |
| Case/N | 208/522 | 185/523 | 187/523 | 202/522 |  |  |  |
| Model 1  OR (95% CI) | 1.00 (Reference) | 0.75 (0.49-1.15) | 0.68 (0.50-0.94) | 0.90 (0.61-1.33) | 0.644 | 0.98 (0.93-1.02) | 0.366 |
| Model 2  OR (95% CI) | 1.00 (Reference) | 0.88 (0.58-1.34) | 0.97 (0.67-1.39) | 1.58 (0.86-2.88) | 0.097 | 1.05 (0.96-1.14) | 0.279 |
| Model 3  OR (95% CI) | 1.00 (Reference) | 0.90 (0.58-1.37) | 1.00 (0.69-1.44) | 1.61 (0.89-2.91) | 0.096 | 1.05 (0.96-1.14) | 0.271 |
| **AF** |  |  |  |  |  |  |  |
| Case/N | 174/522 | 155/523 | 151/523 | 105/522 |  |  |  |
| Model 1  OR(95% CI) | 1.00 (Reference) | 0.71 (0.51-0.98) | 0.68 (0.52-0.87) | 0.51 (0.35-0.74) | 0.002 | 0.93 (0.89-0.97) | 0.001 |
| Model 2  OR (95% CI) | 1.00 (Reference) | 0.87 (0.57-1.32) | 1.15 (0.73-1.81) | 1.15 (0.55-2.38) | 0.618 | 1.05 (0.95-1.15) | 0.361 |
| Model 3  OR (95% CI) | 1.00 (Reference) | 0.87 (0.57-1.34) | 1.14 (0.72-1.83) | 1.11 (0.52-2.33) | 0.706 | 1.05 (0.95-1.15) | 0.398 |
| **DSST** |  |  |  |  |  |  |  |
| Case/N | 163/522 | 143/523 | 132/523 | 90/522 |  |  |  |
| Model 1  OR (95% CI) | 1.00 (Reference) | 0.67 (0.45-0.99) | 0.54 (0.39-0.76) | 0.29 (0.18-0.47) | <0.001 | 0.86 (0.82-0.91) | <0.001 |
| Model 2  OR (95% CI) | 1.00 (Reference) | 0.92 (0.60-1.40) | 1.24 (0.76-2.02) | 1.05 (0.52-2.13) | 0.791 | 1.03 (0.93-1.13) | 0.631 |
| Model 3  OR (95% CI) | 1.00 (Reference) | 0.91 (0.61-1.36) | 1.21 (0.73-1.99) | 0.93 (0.47-1.86) | 0.948 | 1.02 (0.92-1.13) | 0.737 |

Model 1: adjusted for age, gender, ethnicity

Model 2: additionally adjusted for income, education, exercise, current smoker, current drinker, supplement use, BMI, total intake of energy, AHEI, dietary fiber intake

Model 3: additionally adjusted for self-reported of diabetes, hypertension, cardiovascular diseases (congestive heart failure, coronary heart disease, angina, heart attack, and stroke), cancer, and sleep disorders.

BMI, body mass index; AHEI, alternative healthy eating index; Q, quartile; CERAD-WL, Consortium to Establish a Registry for Alzheimer’s Disease Word List Learning Test; CERAD-DR, CERAD Word List Delayed Recall Test; AF, Animal Fluency; DSST, Digit Symbol Substitution Test.

**Supplementary Table 13 Association of dietary carbohydrates with poor cognitive performance among participants whose daily fasting duration was more than 16 hours.**

| **Cognitive Test** | Quartiles of percentage energy from carbohydrates | | | | *P* _for trend_ | Per 10% increase of percentage energy from carbohydrates | *P* value |
| --- | --- | --- | --- | --- | --- | --- | --- |
|  | Q1 (N = 99) | Q2 (N = 99) | Q3 (N = 99) | Q4 (N = 98) |  |  |  |
| **CERAD-WL** |  |  |  |  |  |  |  |
| Case/N | 26/99 | 28/99 | 22/99 | 28/98 |  |  |  |
| Model 1  OR (95% CI) | 1.00 (Reference) | 0.88 (0.39-1.95) | 0.83 (0.33-2.07) | 0.85 (0.38-1.86) | 0.734 | 0.97 (0.87-1.08) | 0.617 |
| Model 2  OR (95% CI) | 1.00 (Reference) | 1.73 (0.67-4.44) | 2.37 (0.61-9.09) | 3.63 (0.84-15.65) | 0.129 | 1.19 (0.98-1.44) | 0.096 |
| Model 3  OR (95% CI) | 1.00 (Reference) | 1.68 (0.66-4.25) | 2.21 (0.56-8.66) | 3.34 (0.73-15.14) | 0.182 | 1.17 (0.96-1.43) | 0.140 |
| **CERAD-DR** |  |  |  |  |  |  |  |
| Case/N | 31/99 | 25/99 | 37/99 | 31/98 |  |  |  |
| Model 1  OR (95% CI) | 1.00 (Reference) | 0.57 (0.25-1.30) | 1.48 (0.73-3.01) | 0.68 (0.27-1.72) | 0.850 | 1.01 (0.90-1.13) | 0.887 |
| Model 2  OR (95% CI) | 1.00 (Reference) | 0.87 (0.33-2.27) | 2.90 (1.00-8.40) | 1.67 (0.43-6.51) | 0.266 | 1.18 (0.99-1.40) | 0.068 |
| Model 3  OR (95% CI) | 1.00 (Reference) | 0.84 (0.31-2.27) | 2.75 (0.92-8.22) | 1.76 (0.45-6.89) | 0.206 | 1.19 (0.99-1.39) | 0.052 |
| **AF** |  |  |  |  |  |  |  |
| Case/N | 36/99 | 35/99 | 26/99 | 15/98 |  |  |  |
| Model 1  OR (95% CI) | 1.00 (Reference) | 0.95 (0.42-2.13) | 0.54 (0.28-1.03) | 0.38 (0.16-0.88) | 0.007 | 0.87 (0.79-0.96) | 0.011 |
| Model 2  OR (95% CI) | 1.00 (Reference) | 2.13 (0.95-4.76) | 1.61 (0.60-4.32) | 2.05 (0.54-7.74) | 0.515 | 1.06 (0.88-1.28) | 0.549 |
| Model 3  OR (95% CI) | 1.00 (Reference) | 2.00 (0.95-4.20) | 1.43 (0.55-3.71) | 1.60 (0.43-5.92) | 0.749 | 1.02 (0.84-1.23) | 0.834 |
| **DSST** |  |  |  |  |  |  |  |
| Case/N | 38/99 | 22/99 | 29/99 | 18/98 |  |  |  |
| Model 1  OR (95% CI) | 1.00 (Reference) | 0.38 (0.18-0.76) | 0.48 (0.23-1.01) | 0.25 (0.11-0.61) | 0.007 | 0.88 (0.79-0.97) | 0.014 |
| Model 2  OR (95% CI) | 1.00 (Reference) | 0.51 (0.15-1.66) | 0.70 (0.15-3.29) | 0.61 (0.04-8.91) | 0.818 | 1.07 (0.82-1.39) | 0.632 |
| Model 3  OR (95% CI) | 1.00 (Reference) | 0.49 (0.16-1.48) | 0.73 (0.16-3.36) | 0.57 (0.03-9.39) | 0.781 | 1.05 (0.81-1.36) | 0.718 |

Model 1: adjusted for age, gender, ethnicity

Model 2: additionally adjusted for income, education, exercise, current smoker, current drinker, supplement use, BMI, total intake of energy, AHEI, dietary fiber intake

Model 3: additionally adjusted for self-reported of diabetes, hypertension, cardiovascular diseases (congestive heart failure, coronary heart disease, angina, heart attack, and stroke), cancer, and sleep disorders.

BMI, body mass index; AHEI, alternative healthy eating index; Q, quartile; CERAD-WL, Consortium to Establish a Registry for Alzheimer’s Disease Word List Learning Test; CERAD-DR, CERAD Word List Delayed Recall Test; AF, Animal Fluency; DSST, Digit Symbol Substitution Test.

**Supplementary Table 14 Association of dietary carbohydrates with poor cognitive performance among total participants.**

| **Cognitive Test** | Quartiles of percentage energy from carbohydrates | | | | *P* _for trend_ | *P* _for interaction with fasting duration_ | Per 10% increase of percentage energy from carbohydrates | *P* value |
| --- | --- | --- | --- | --- | --- | --- | --- | --- |
|  | Q1 (N = 621) | Q2 (N = 621) | Q3 (N = 621) | Q4 (N = 622) |  |  |  |  |
| **Total score** |  |  |  |  |  |  |  |  |
| Case/N | 123/621 | 132/621 | 172/621 | 205/622 |  |  |  |  |
| Model 1  OR (95% CI) | 1.00 (Reference) | 0.92 (0.65-1.31) | 1.26 (0.95-1.68) | 1.73 (1.19-2.52) | 0.002 | 0.169 | 1.08 (1.04-1.13) | <0.001 |
| Model 2  OR (95% CI) | 1.00 (Reference) | 1.08 (0.72-1.61) | 1.15 (0.79-1.66) | 1.19 (0.78-1.81) | 0.364 | 0.623 | 1.02 (0.98-1.07) | 0.238 |
| Model 3  OR (95% CI) | 1.00 (Reference) | 1.04 (0.69-1.57) | 1.12 (0.78-1.63) | 1.14 (0.74-1.76) | 0.471 | 0.494 | 1.02 (0.97-1.07) | 0.313 |

Model 1: adjusted for age, gender, ethnicity

Model 2: additionally adjusted for income, education, exercise, current smoker, current drinker, supplement use, BMI, total intake of energy, AHEI, dietary fiber intake

Model 3: additionally adjusted for self-reported of diabetes, hypertension, cardiovascular diseases (congestive heart failure, coronary heart disease, angina, heart attack, and stroke), cancer, and sleep disorders.

BMI, body mass index; AHEI, alternative healthy eating index; Q, quartile; CERAD-WL, Consortium to Establish a Registry for Alzheimer’s Disease Word List Learning Test; CERAD-DR, CERAD Word List Delayed Recall Test; AF, Animal Fluency; DSST, Digit Symbol Substitution Test.

**Supplementary Table 15 Association of dietary carbohydrates with poor cognitive performance among participants whose daily fasting duration was fewer than 16 hours.**

| **Cognitive Test** | Quartiles of percentage energy from carbohydrates | | | | *P* _for trend_ | Per 10% increase of percentage energy from carbohydrates | *P* value |
| --- | --- | --- | --- | --- | --- | --- | --- |
|  | Q1 (N = 523) | Q2 (N = 522) | Q3 (N = 522) | Q4 (N = 523) |  |  |  |
| **Total score** |  |  |  |  |  |  |  |
| Case/N | 104/523 | 113/522 | 146/522 | 173/523 |  |  |  |
| Model 1  OR (95% CI) | 1.00 (Reference) | 1.10 (0.80-1.51) | 1.45 (1.02-2.08) | 2.00 (1.40-2.86) | <0.001 | 1.10 (1.05-1.15) | <0.001 |
| Model 2  OR (95% CI) | 1.00 (Reference) | 1.27 (0.85-1.92) | 1.36 (0.85-2.17) | 1.58 (1.06-2.36) | 0.028 | 1.06 (1.01-1.11) | 0.016 |
| Model 3  OR (95% CI) | 1.00 (Reference) | 1.21 (0.80-1.84) | 1.30 (0.81-2.11) | 1.51 (1.01-2.27) | 0.052 | 1.05 (1.01-1.10) | 0.032 |

Model 1: adjusted for age, gender, ethnicity

Model 2: additionally adjusted for income, education, exercise, current smoker, current drinker, supplement use, BMI, total intake of energy, AHEI, dietary fiber intake

Model 3: additionally adjusted for self-reported of diabetes, hypertension, cardiovascular diseases (congestive heart failure, coronary heart disease, angina, heart attack, and stroke), cancer, and sleep disorders.

BMI, body mass index; AHEI, alternative healthy eating index; Q, quartile; CERAD-WL, Consortium to Establish a Registry for Alzheimer’s Disease Word List Learning Test; CERAD-DR, CERAD Word List Delayed Recall Test; AF, Animal Fluency; DSST, Digit Symbol Substitution Test.

**Supplementary Table 16 Association of dietary carbohydrates with poor cognitive performance among participants whose daily fasting duration was more than 16 hours.**

| **Cognitive Test** | Quartiles of percentage energy from carbohydrates | | | | *P* _for trend_ | Per 10% increase of percentage energy from carbohydrates | *P* value |
| --- | --- | --- | --- | --- | --- | --- | --- |
|  | Q1 (N = 99) | Q2 (N = 99) | Q3 (N = 99) | Q4 (N = 98) |  |  |  |
| **CERAD-WL** |  |  |  |  |  |  |  |
| Case/N | 20/99 | 25/99 | 25/99 | 31/98 |  |  |  |
| Model 1  OR (95% CI) | 1.00 (Reference) | 0.95 (0.39-2.29) | 0.86 (0.36-2.04) | 1.49 (0.57-3.87) | 0.517 | 1.03 (0.90-1.18) | 0.586 |
| Model 2  OR(95% CI) | 1.00 (Reference) | 0.91 (0.29-2.79) | 0.50 (0.14-1.77) | 0.78 (0.21-2.92) | 0.560 | 0.94 (0.79-1.13) | 0.570 |
| Model 3  OR(95% CI) | 1.00 (Reference) | 0.65 (0.22-1.92) | 0.41 (0.11-1.47) | 0.61 (0.15-2.37) | 0.445 | 0.92 (0.76-1.11) | 0.428 |

Model 1: adjusted for age, gender, ethnicity

Model 2: additionally adjusted for income, education, exercise, current smoker, current drinker, supplement use, BMI, total intake of energy, AHEI, dietary fiber intake

Model 3: additionally adjusted for self-reported of diabetes, hypertension, cardiovascular diseases (congestive heart failure, coronary heart disease, angina, heart attack, and stroke), cancer, and sleep disorders.

BMI, body mass index; AHEI, alternative healthy eating index; Q, quartile; CERAD-WL, Consortium to Establish a Registry for Alzheimer’s Disease Word List Learning Test; CERAD-DR, CERAD Word List Delayed Recall Test; AF, Animal Fluency; DSST, Digit Symbol Substitution Test.

**Supplementary Table 17 Association of dietary carbohydrates with poor cognitive performance among male participants.**

| **Cognitive Test** | Quartiles of percentage energy from carbohydrates | | | | *P* _for trend_ | Per 10% increase of percentage energy from carbohydrates | *P* value |
| --- | --- | --- | --- | --- | --- | --- | --- |
|  | Q1 (N = 300) | Q2 (N = 299) | Q3 (N = 301) | Q4 (N = 299) |  |  |  |
| **CERAD-WL** |  |  |  |  |  |  |  |
| Case/N | 87/300 | 85/299 | 119/301 | 112/299 |  |  |  |
| Model 1  OR (95% CI) | 1.00 (Reference) | 0.81 (0.50-1.31) | 1.38 (0.96-1.98) | 1.79 (1.20-2.67) | <0.001 | 1.08 (1.03-1.14) | 0.002 |
| Model 2  OR (95% CI) | 1.00 (Reference) | 0.90 (0.56-1.47) | 1.46 (0.99-2.14) | 1.81 (1.09-3.01) | 0.011 | 1.08 (1.02-1.15) | 0.020 |
| Model 3  OR (95% CI) | 1.00 (Reference) | 0.93 (0.57-1.52) | 1.53 (1.03-2.30) | 1.87 (1.14-3.05) | 0.008 | 1.09 (1.02-1.16) | 0.015 |
| **CERAD-DR** |  |  |  |  |  |  |  |
| Case/N | 127/300 | 124/299 | 159/301 | 149/299 |  |  |  |
| Model 1  OR (95% CI) | 1.00 (Reference) | 1.11 (0.80-1.53) | 1.05 (0.75-1.48) | 1.30 (0.86-1.97) | 0.212 | 1.02 (0.97-1.06) | 0.327 |
| Model 2  OR (95% CI) | 1.00 (Reference) | 1.18 (0.84-1.64) | 1.08 (0.73-1.59) | 1.23 (0.77-1.95) | 0.424 | 1.01 (0.96-1.07) | 0.614 |
| Model 3  OR (95% CI) | 1.00 (Reference) | 1.14 (0.79-1.64) | 1.08 (0.74-1.59) | 1.23 (0.78-1.96) | 0.381 | 1.01 (0.96-1.07) | 0.573 |
| **AF** |  |  |  |  |  |  |  |
| Case/N | 79/300 | 81/299 | 89/301 | 99/299 |  |  |  |
| Model 1  OR (95% CI) | 1.00 (Reference) | 0.80 (0.43-1.48) | 0.85 (0.51-1.43) | 1.15 (0.65-2.03) | 0.003 | 1.02 (0.95-1.08) | 0.552 |
| Model 2  OR (95% CI) | 1.00 (Reference) | 0.95 (0.47-1.93) | 0.85 (0.45-1.59) | 1.08 (0.54-2.16) | 0.918 | 1.01 (0.93-1.09) | 0.851 |
| Model 3  OR (95% CI) | 1.00 (Reference) | 0.99 (0.48-2.05) | 0.90 (0.47-1.74) | 1.11 (0.55-2.24) | 0.403 | 1.02 (0.93-1.09) | 0.780 |
| **DSST** |  |  |  |  |  |  |  |
| Case/N | 70/300 | 71/299 | 90/301 | 109/299 |  |  |  |
| Model 1  OR (95% CI) | 1.00 (Reference) | 0.82 (0.53-1.26) | 1.19 (0.74-1.89) | 1.70 (0.98-2.93) | 0.055 | 1.07 (0.99-1.16) | 0.068 |
| Model 2  OR(95% CI) | 1.00 (Reference) | 1.07 (0.66-1.73) | 1.13 (0.63-2.03) | 1.53 (0.90-2.61) | 0.150 | 1.04 (0.97-1.12) | 0.237 |
| Model 3  OR(95% CI) | 1.00 (Reference) | 0.97 (0.60-1.58) | 1.03 (0.56-1.88) | 1.49 (0.86-2.60) | 0.197 | 1.04 (0.96-1.12) | 0.279 |

Model 1: adjusted for age, ethnicity

Model 2: additionally adjusted for income, education, exercise, current smoker, current drinker, supplement use, BMI, total intake of energy, AHEI, dietary fiber intake

Model 3: additionally adjusted for self-reported of diabetes, hypertension, cardiovascular diseases (congestive heart failure, coronary heart disease, angina, heart attack, and stroke), cancer, and sleep disorders.

BMI, body mass index; AHEI, alternative healthy eating index; Q, quartile; CERAD-WL, Consortium to Establish a Registry for Alzheimer’s Disease Word List Learning Test; CERAD-DR, CERAD Word List Delayed Recall Test; AF, Animal Fluency; DSST, Digit Symbol Substitution Test.

**Supplementary Table 18 Association of dietary carbohydrates with poor cognitive performance among female participants.**

| **Cognitive Test** | Quartiles of percentage energy from carbohydrates | | | | *P* _for trend_ | Per 10% increase of percentage energy from carbohydrates | *P* value |
| --- | --- | --- | --- | --- | --- | --- | --- |
|  | Q1 (N = 322) | Q2 (N = 321) | Q3 (N = 322) | Q4 (N = 321) |  |  |  |
| **CERAD-WL** |  |  |  |  |  |  |  |
| Case/N | 40/322 | 64/321 | 74/322 | 94/321 |  |  |  |
| Model 1  OR (95% CI) | 1.00 (Reference) | 1.68 (1.03-2.73) | 1.51 (0.90-2.54) | 2.42 (1.51-3.86) | 0.003 | 1.12 (1.05-1.19) | 0.001 |
| Model 2  OR (95% CI) | 1.00 (Reference) | 1.85 (1.04-3.27) | 1.22 (0.64-2.32) | 1.74 (0.93-3.27) | 0.204 | 1.07 (0.99-1.16) | 0.101 |
| Model 3  OR (95% CI) | 1.00 (Reference) | 1.92 (1.06-3.50) | 1.23 (0.64-2.35) | 1.73 (0.89-3.34) | 0.242 | 1.07 (0.98-1.16) | 0.122 |
| **CERAD-DR** |  |  |  |  |  |  |  |
| Case/N | 80/322 | 91/321 | 123/322 | 120/321 |  |  |  |
| Model 1  OR (95% CI) | 1.00 (Reference) | 1.19 (0.73-1.94) | 1.63 (1.01-2.64) | 1.76 (1.07-2.92) | 0.024 | 1.07 (1.01-1.14) | 0.028 |
| Model 2  OR (95% CI) | 1.00 (Reference) | 1.20 (0.72-2.00) | 1.44 (0.86-2.40) | 1.39 (0.81-2.37) | 0.191 | 1.04 (0.97-1.11) | 0.255 |
| Model 3  OR (95% CI) | 1.00 (Reference) | 1.22 (0.71-2.09) | 1.43 (0.87-2.37) | 1.37 (0.78-2.40) | 0.220 | 1.04 (0.97-1.11) | 0.281 |
| **AF** |  |  |  |  |  |  |  |
| Case/N | 67/322 | 95/321 | 103/322 | 116/321 |  |  |  |
| Model 1  OR (95% CI) | 1.00 (Reference) | 1.32 (0.81-2.17) | 1.40 (0.89-2.19) | 1.76 (1.24-2.49) | 0.004 | 1.06 (1.01-1.12) | 0.010 |
| Model 2  OR (95% CI) | 1.00 (Reference) | 1.37 (0.76-2.45) | 1.23 (0.72-2.10) | 1.33 (0.78-2.25) | 0.336 | 1.02 (0.96-1.09) | 0.407 |
| Model 3  OR (95% CI) | 1.00 (Reference) | 1.43 (0.78-2.64) | 1.26 (0.74-2.15) | 1.30 (0.74-2.28) | 0.406 | 1.02 (0.96-1.09) | 0.478 |
| **DSST** |  |  |  |  |  |  |  |
| Case/N | 43/322 | 61/321 | 85/322 | 94/321 |  |  |  |
| Model 1  OR (95% CI) | 1.00 (Reference) | 1.51 (0.94-2.44) | 2.04 (1.33-3.11) | 2.45 (1.65-3.65) | <0.001 | 1.10 (1.05-1.16) | <0.001 |
| Model 2  OR(95% CI) | 1.00 (Reference) | 1.76 (0.98-3.17) | 1.63 (0.91-2.92) | 1.42 (0.71-2.83) | 0.367 | 1.02 (0.94-1.10) | 0.593 |
| Model 3  OR(95% CI) | 1.00 (Reference) | 1.90 (0.98-3.70) | 1.71 (0.94-3.09) | 1.36 (0.66-2.80) | 0.451 | 1.01 (0.93-1.10) | 0.696 |

Model 1: adjusted for age, ethnicity

Model 2: additionally adjusted for income, education, exercise, current smoker, current drinker, supplement use, BMI, total intake of energy, AHEI, dietary fiber intake

Model 3: additionally adjusted for self-reported of diabetes, hypertension, cardiovascular diseases (congestive heart failure, coronary heart disease, angina, heart attack, and stroke), cancer, and sleep disorders.

BMI, body mass index; AHEI, alternative healthy eating index; Q, quartile; CERAD-WL, Consortium to Establish a Registry for Alzheimer’s Disease Word List Learning Test; CERAD-DR, CERAD Word List Delayed Recall Test; AF, Animal Fluency; DSST, Digit Symbol Substitution Test.

**Supplementary Table 19 Association of dietary carbohydrates with poor cognitive performance among Other American participants.**

| **Cognitive Test** | Quartiles of percentage energy from carbohydrates | | | | *P* _for trend_ | Per 10% increase of percentage energy from carbohydrates | *P* value |
| --- | --- | --- | --- | --- | --- | --- | --- |
|  | Q1 (N = 167) | Q2 (N = 167) | Q3 (N = 167) | Q4 (N = 168) |  |  |  |
| **CERAD-WL** |  |  |  |  |  |  |  |
| Case/N | 46/167 | 53/167 | 53/167 | 63/168 |  |  |  |
| Model 1  OR (95% CI) | 1.00 (Reference) | 1.46 (0.97-2.21) | 1.56 (0.89-2.75) | 2.54 (1.29-4.99) | 0.010 | 1.12 (1.03-1.22) | 0.007 |
| Model 2  OR (95% CI) | 1.00 (Reference) | 1.27 (0.87-1.86) | 1.39 (0.76-2.55) | 1.70 (0.82-3.51) | 0.162 | 1.07 (0.98-1.17) | 0.130 |
| Model 3  OR (95% CI) | 1.00 (Reference) | 1.30 (0.86-1.97) | 1.33 (0.69-2.56) | 1.67 (0.81-3.47) | 0.198 | 1.06 (0.97-1.16) | 0.181 |
| **CERAD-DR** |  |  |  |  |  |  |  |
| Case/N | 56/167 | 64/167 | 69/167 | 75/168 |  |  |  |
| Model 1  OR (95% CI) | 1.00 (Reference) | 1.34 (0.88-2.04) | 2.16 (1.07-4.35) | 2.29 (1.22-4.31) | 0.015 | 1.11 (1.03-1.20) | 0.009 |
| Model 2  OR (95% CI) | 1.00 (Reference) | 1.33 (0.85-2.08) | 2.12 (1.11-4.03) | 1.84 (0.90-3.74) | 0.072 | 1.09 (1.00-1.18) | 0.052 |
| Model 3  OR (95% CI) | 1.00 (Reference) | 1.38 (0.89-2.13) | 2.19 (1.14-4.18) | 1.83 (0.92-3.62) | 0.070 | 1.09 (1.01-1.18) | 0.051 |
| **AF** |  |  |  |  |  |  |  |
| Case/N | 50/167 | 57/167 | 57/167 | 68/168 |  |  |  |
| Model 1  OR (95% CI) | 1.00 (Reference) | 1.48 (0.94-2.30) | 1.21 (0.82-1.78) | 1.56 (0.94-2.60) | 0.179 | 1.04 (0.97-1.10) | 0.240 |
| Model 2  OR (95% CI) | 1.00 (Reference) | 1.34 (0.81-2.20) | 1.13 (0.74-1.72) | 1.17 (0.64-2.13) | 0.744 | 1.00 (0.93-1.08) | 0.886 |
| Model 3  OR (95% CI) | 1.00 (Reference) | 1.38 (0.82-2.30) | 1.18 (0.77-1.80) | 1.20 (0.66-2.17) | 0.678 | 1.01 (0.93-1.09) | 0.783 |
| **DSST** |  |  |  |  |  |  |  |
| Case/N | 44/167 | 56/167 | 50/167 | 78/168 |  |  |  |
| Model 1  OR (95% CI) | 1.00 (Reference) | 1.46 (1.01-2.10) | 1.18 (0.72-1.93) | 2.40 (1.43-4.04) | 0.006 | 1.10 (1.03-1.18) | 0.011 |
| Model 2  OR(95% CI) | 1.00 (Reference) | 1.33 (0.74-2.40) | 1.06 (0.52-2.16) | 1.74 (0.77-3.93) | 0.271 | 1.05 (0.94-1.17) | 0.356 |
| Model 3  OR(95% CI) | 1.00 (Reference) | 1.30 (0.72-2.35) | 1.06 (0.52-2.15) | 1.71 (0.76-3.80) | 0.278 | 1.05 (0.94-1.17) | 0.365 |

Model 1: adjusted for age, gender

Model 2: additionally adjusted for income, education, exercise, current smoker, current drinker, supplement use, BMI, total intake of energy, AHEI, dietary fiber intake

Model 3: additionally adjusted for self-reported of diabetes, hypertension, cardiovascular diseases (congestive heart failure, coronary heart disease, angina, heart attack, and stroke), cancer, and sleep disorders.

BMI, body mass index; AHEI, alternative healthy eating index; Q, quartile; CERAD-WL, Consortium to Establish a Registry for Alzheimer’s Disease Word List Learning Test; CERAD-DR, CERAD Word List Delayed Recall Test; AF, Animal Fluency; DSST, Digit Symbol Substitution Test.

**Supplementary Table 20 Association of dietary carbohydrates with poor cognitive performance among Non-Hispanic Black participants.**

| **Cognitive Test** | Quartiles of percentage energy from carbohydrates | | | | *P* _for trend_ | Per 10% increase of percentage energy from carbohydrates | *P* value |
| --- | --- | --- | --- | --- | --- | --- | --- |
|  | Q1 (N = 141) | Q2 (N = 140) | Q3 (N = 141) | Q4 (N = 142) |  |  |  |
| **CERAD-WL** |  |  |  |  |  |  |  |
| Case/N | 33/141 | 34/140 | 34/141 | 48/142 |  |  |  |
| Model 1  OR (95% CI) | 1.00 (Reference) | 1.17 (0.65-2.08) | 1.07 (0.59-1.93) | 1.84 (1.05-3.24) | 0.047 | 1.08 (1.01-1.16) | 0.036 |
| Model 2  OR (95% CI) | 1.00 (Reference) | 1.36 (0.78-2.35) | 1.16 (0.60-2.26) | 2.12 (1.20-3.75) | 0.030 | 1.10 (1.02-1.19) | 0.025 |
| Model 3  OR (95% CI) | 1.00 (Reference) | 1.32 (0.76-2.30) | 1.18 (0.59-2.35) | 2.13 (1.21-3.72) | 0.032 | 1.10 (1.02-1.19) | 0.030 |
| **CERAD-DR** |  |  |  |  |  |  |  |
| Case/N | 55/141 | 49/140 | 65/141 | 61/142 |  |  |  |
| Model 1  OR (95% CI) | 1.00 (Reference) | 0.80 (0.47-1.36) | 1.28 (0.75-2.16) | 1.06 (0.64-1.75) | 0.516 | 1.01 (0.95-1.08) | 0.608 |
| Model 2  OR (95% CI) | 1.00 (Reference) | 0.92 (0.53-1.58) | 1.43 (0.83-2.49) | 1.20 (0.78-1.82) | 0.232 | 1.03 (0.97-1.10) | 0.280 |
| Model 3  OR (95% CI) | 1.00 (Reference) | 0.93 (0.54-1.62) | 1.48 (0.85-2.58) | 1.21 (0.79-1.87) | 0.224 | 1.03 (0.97-1.10) | 0.263 |
| **AF** |  |  |  |  |  |  |  |
| Case/N | 54/141 | 53/140 | 65/141 | 67/142 |  |  |  |
| Model 1  OR (95% CI) | 1.00 (Reference) | 1.08 (0.71-1.64) | 1.48 (0.96-2.28) | 1.31 (0.81-2.10) | 0.156 | 1.03 (0.98-1.08) | 0.195 |
| Model 2  OR (95% CI) | 1.00 (Reference) | 1.30 (0.77-2.19) | 1.52 (0.94-2.48) | 1.18 (0.70-1.98) | 0.441 | 1.01 (0.96-1.07) | 0.560 |
| Model 3  OR (95% CI) | 1.00 (Reference) | 1.28 (0.73-2.22) | 1.53 (0.91-2.59) | 1.16 (0.69-1.95) | 0.463 | 1.01 (0.96-1.07) | 0.575 |
| **DSST** |  |  |  |  |  |  |  |
| Case/N | 49/141 | 43/140 | 54/141 | 59/142 |  |  |  |
| Model 1  OR (95% CI) | 1.00 (Reference) | 0.80 (0.52-1.22) | 1.02 (0.60-1.74) | 0.97 (0.55-1.68) | 0.927 | 0.99 (0.92-1.06) | 0.821 |
| Model 2  OR(95% CI) | 1.00 (Reference) | 0.94 (0.50-1.76) | 1.00 (0.54-1.87) | 0.82 (0.44-1.53) | 0.588 | 0.96 (0.88-1.05) | 0.436 |
| Model 3  OR(95% CI) | 1.00 (Reference) | 0.91 (0.49-1.70) | 1.05 (0.57-1.94) | 0.80 (0.41-1.56) | 0.603 | 0.96 (0.87-1.05) | 0.470 |

Model 1: adjusted for age, gender

Model 2: additionally adjusted for income, education, exercise, current smoker, current drinker, supplement use, BMI, total intake of energy, AHEI, dietary fiber intake

Model 3: additionally adjusted for self-reported of diabetes, hypertension, cardiovascular diseases (congestive heart failure, coronary heart disease, angina, heart attack, and stroke), cancer, and sleep disorders.

BMI, body mass index; AHEI, alternative healthy eating index; Q, quartile; CERAD-WL, Consortium to Establish a Registry for Alzheimer’s Disease Word List Learning Test; CERAD-DR, CERAD Word List Delayed Recall Test; AF, Animal Fluency; DSST, Digit Symbol Substitution Test.

**Supplementary Table 21 Association of dietary carbohydrates with poor cognitive performance among Non-Hispanic White participants.**

| **Cognitive Test** | Quartiles of percentage energy from carbohydrates | | | | *P* _for trend_ | Per 10% increase of percentage energy from carbohydrates | *P* value |
| --- | --- | --- | --- | --- | --- | --- | --- |
|  | Q1 (N = 312) | Q2 (N = 314) | Q3 (N = 313) | Q4 (N = 312) |  |  |  |
| **CERAD-WL** |  |  |  |  |  |  |  |
| Case/N | 60/312 | 62/314 | 90/313 | 99/312 |  |  |  |
| Model 1  OR (95% CI) | 1.00 (Reference) | 0.99 (0.58-1.69) | 1.19 (0.86-1.67) | 1.78 (1.18-2.68) | 0.002 | 1.08 (1.03-1.13) | 0.002 |
| Model 2  OR (95% CI) | 1.00 (Reference) | 1.07 (0.62-1.84) | 1.26 (0.87-0.83) | 0.71 (1.01-2.87) | 0.031 | 1.07 (1.01-1.14) | 0.020 |
| Model 3  OR (95% CI) | 1.00 (Reference) | 1.01 (0.56-1.80) | 1.22 (0.83-1.79) | 1.67 (0.97-2.85) | 0.043 | 1.07 (1.01-1.14) | 0.028 |
| **CERAD-DR** |  |  |  |  |  |  |  |
| Case/N | 107/312 | 106/314 | 132/313 | 133/312 |  |  |  |
| Model 1  OR (95% CI) | 1.00 (Reference) | 1.00 (0.73-1.37) | 1.07 (0.79-1.45) | 1.27 (0.87-1.86) | 0.187 | 1.03 (0.98-1.07) | 0.158 |
| Model 2  OR (95% CI) | 1.00 (Reference) | 1.06 (0.77-1.44) | 1.11 (0.81-1.53) | 1.18 (0.80-1.75) | 0.350 | 1.02 (0.97-1.07) | 0.326 |
| Model 3  OR (95% CI) | 1.00 (Reference) | 1.03 (0.73-1.45) | 1.11 (0.81-1.53) | 1.18 (0.80-1.75) | 0.327 | 1.02 (0.98-1.07) | 0.310 |
| **AF** |  |  |  |  |  |  |  |
| Case/N | 47/312 | 58/314 | 76/313 | 77/312 |  |  |  |
| Model 1  OR (95% CI) | 1.00 (Reference) | 0.84 (0.45-1.54) | 0.98 (0.62-1.54) | 1.15 (0.80-1.65) | 0.310 | 1.01 (0.98-1.04) | 0.352 |
| Model 2  OR (95% CI) | 1.00 (Reference) | 0.90 (0.44-1.86) | 1.01 (0.59-1.74) | 1.09 (0.65-1.83) | 0.651 | 1.01 (0.96-1.05) | 0.783 |
| Model 3  OR (95% CI) | 1.00 (Reference) | 0.86 (0.41-1.78) | 0.98 (0.57-1.69) | 1.07 (0.62-1.84) | 0.719 | 1.00 (0.96-1.05) | 0.860 |
| **DSST** |  |  |  |  |  |  |  |
| Case/N | 32/312 | 43/314 | 55/313 | 60/312 |  |  |  |
| Model 1  OR (95% CI) | 1.00 (Reference) | 1.09 (0.76-1.56) | 1.09 (0.71-1.65) | 1.41 (0.88-2.27) | 0.191 | 1.03 (0.97-1.10) | 0.265 |
| Model 2  OR(95% CI) | 1.00 (Reference) | 1.34 (0.83-2.16) | 1.13 (0.69-1.86) | 1.25 (0.69-2.24) | 0.547 | 1.01 (0.94-1.08) | 0.766 |
| Model 3  OR(95% CI) | 1.00 (Reference) | 1.15 (0.72-1.84) | 1.03 (0.61-1.72) | 1.12 (0.62-2.04) | 0.781 | 1.00 (0.92-1.08) | 0.998 |

Model 1: adjusted for age, gender

Model 2: additionally adjusted for income, education, exercise, current smoker, current drinker, supplement use, BMI, total intake of energy, AHEI, dietary fiber intake

Model 3: additionally adjusted for self-reported of diabetes, hypertension, cardiovascular diseases (congestive heart failure, coronary heart disease, angina, heart attack, and stroke), cancer, and sleep disorders.

BMI, body mass index; AHEI, alternative healthy eating index; Q, quartile; CERAD-WL, Consortium to Establish a Registry for Alzheimer’s Disease Word List Learning Test; CERAD-DR, CERAD Word List Delayed Recall Test; AF, Animal Fluency; DSST, Digit Symbol Substitution Test.

**Supplementary Table 22 Association of dietary carbohydrates with poor cognitive performance among participants with regular exercise.**

| **Cognitive Test** | Quartiles of percentage energy from carbohydrates | | | | *P* _for trend_ | Per 10% increase of percentage energy from carbohydrates | *P* value |
| --- | --- | --- | --- | --- | --- | --- | --- |
|  | Q1 (N = 140) | Q2 (N = 141) | Q3 (N = 139) | Q4 (N = 141) |  |  |  |
| **CERAD-WL** |  |  |  |  |  |  |  |
| Case/N | 22/140 | 19/141 | 24/139 | 40/141 |  |  |  |
| Model 1  OR (95% CI) | 1.00 (Reference) | 0.98 (0.31-3.01) | 0.99 (0.47-2.07) | 2.84 (1.28-6.32) | 0.011 | 1.13 (1.02-1.24) | 0.020 |
| Model 2  OR (95% CI) | 1.00 (Reference) | 0.88 (0.29-2.64) | 1.02 (0.44-2.36) | 3.21 (1.42-7.26) | 0.010 | 1.15 (1.03-1.28) | 0.019 |
| Model 3  OR (95% CI) | 1.00 (Reference) | 0.79 (0.24-2.50) | 0.99 (0.44-2.21) | 3.05 (1.31-7.10) | 0.015 | 1.15 (1.03-1.28) | 0.024 |
| **CERAD-DR** |  |  |  |  |  |  |  |
| Case/N | 39/140 | 37/141 | 44/139 | 53/141 |  |  |  |
| Model 1  OR (95% CI) | 1.00 (Reference) | 1.17 (0.48-2.85) | 1.13 (0.61-2.08) | 1.94 (1.11-3.42) | 0.010 | 1.04 (0.99-1.11) | 0.111 |
| Model 2  OR (95% CI) | 1.00 (Reference) | 1.07 (0.43-2.67) | 1.26 (0.65-2.45) | 1.80 (0.97-3.36) | 0.044 | 1.04 (0.96-1.12) | 0.270 |
| Model 3  OR (95% CI) | 1.00 (Reference) | 1.04 (0.41-2.68) | 1.28 (0.66-2.48) | 1.85 (0.97-3.54) | 0.043 | 1.04 (0.97-1.13) | 0.252 |
| **AF** |  |  |  |  |  |  |  |
| Case/N | 27/140 | 27/141 | 29/139 | 39/141 |  |  |  |
| Model 1  OR (95% CI) | 1.00 (Reference) | 1.11 (0.44-2.77) | 0.77 (0.34-1.77) | 1.86 (0.80-4.34) | 0.205 | 1.04 (0.95-1.15) | 0.341 |
| Model 2  OR (95% CI) | 1.00 (Reference) | 0.99 (0.39-2.51) | 0.63 (0.25-1.61) | 1.36 (0.61-3.02) | 0.651 | 1.00 (0.89-1.11) | 0.995 |
| Model 3  OR (95% CI) | 1.00 (Reference) | 0.89 (0.35-2.22) | 0.63 (0.24-1.65) | 1.28 (0.57-2.85) | 0.739 | 0.99 (0.89-1.11) | 0.977 |
| **DSST** |  |  |  |  |  |  |  |
| Case/N | 10/140 | 17/141 | 16/139 | 35/141 |  |  |  |
| Model 1  OR (95% CI) | 1.00 (Reference) | 3.14 (1.21-8.14) | 3.13 (1.28-7.60) | 7.52 (3.61-15.65) | <0.001 | 1.21 (1.10-1.33) | <0.001 |
| Model 2  OR(95% CI) | 1.00 (Reference) | 2.99 (1.15-7.78) | 3.19 (1.14-8.92) | 6.09 (2.53-14.68) | 0.003 | 1.18 (1.05-1.33) | 0.008 |
| Model 3  OR(95% CI) | 1.00 (Reference) | 2.86 (1.12-7.33) | 3.10 (1.03-9.32) | 6.63 (2.87-15.29) | 0.002 | 1.19 (1.07-1.34) | 0.006 |

Model 1: adjusted for age, gender, ethnicity

Model 2: additionally adjusted for income, education, current smoker, current drinker, supplement use, BMI, total intake of energy, AHEI, dietary fiber intake

Model 3: additionally adjusted for self-reported of diabetes, hypertension, cardiovascular diseases (congestive heart failure, coronary heart disease, angina, heart attack, and stroke), cancer, and sleep disorders.

BMI, body mass index; AHEI, alternative healthy eating index; Q, quartile; CERAD-WL, Consortium to Establish a Registry for Alzheimer’s Disease Word List Learning Test; CERAD-DR, CERAD Word List Delayed Recall Test; AF, Animal Fluency; DSST, Digit Symbol Substitution Test.

**Supplementary Table 23 Association of dietary carbohydrates with poor cognitive performance among participants with no regular exercise.**

| **Cognitive Test** | Quartiles of percentage energy from carbohydrates | | | | *P* _for trend_ | Per 10% increase of percentage energy from carbohydrates | *P* value |
| --- | --- | --- | --- | --- | --- | --- | --- |
|  | Q1 (N = 385) | Q2 (N = 385) | Q3 (N = 385) | Q4 (N = 385) |  |  |  |
| **CERAD-WL** |  |  |  |  |  |  |  |
| Case/N | 74/385 | 93/385 | 110/385 | 117/385 |  |  |  |
| Model 1  OR (95% CI) | 1.00 (Reference) | 1.26 (0.77-2.06) | 1.69 (1.16-2.45) | 2.46 (1.59-3.81) | <0.001 | 1.12 (1.06-1.18) | <0.001 |
| Model 2  OR (95% CI) | 1.00 (Reference) | 1.48 (0.87-2.50) | 1.93 (1.27-2.92) | 2.50 (1.43-4.37) | 0.001 | 1.12 (1.05-1.21) | 0.002 |
| Model 3  OR (95% CI) | 1.00 (Reference) | 1.47 (0.85-2.54) | 1.88 (1.25-2.84) | 2.47 (1.40-4.33) | 0.003 | 1.12 (1.05-1.20) | 0.003 |
| **CERAD-DR** |  |  |  |  |  |  |  |
| Case/N | 121/385 | 129/385 | 164/385 | 156/385 |  |  |  |
| Model 1  OR (95% CI) | 1.00 (Reference) | 1.14 (0.81-1.60) | 1.53 (1.14-2.06) | 1.69 (1.17-2.43) | 0.002 | 1.06 (1.01-1.11) | 0.017 |
| Model 2  OR (95% CI) | 1.00 (Reference) | 1.22 (0.86-1.72) | 1.62 (1.21-2.15) | 1.71 (1.13-2.59) | 0.006 | 1.06 (1.01-1.12) | 0.041 |
| Model 3  OR (95% CI) | 1.00 (Reference) | 1.22 (0.85-1.75) | 1.65 (1.25-2.18) | 1.72 (1.13-2.61) | 0.007 | 1.06 (1.01-1.11) | 0.041 |
| **AF** |  |  |  |  |  |  |  |
| Case/N | 86/385 | 115/385 | 122/385 | 139/385 |  |  |  |
| Model 1  OR (95% CI) | 1.00 (Reference) | 0.99 (0.59-1.66) | 1.26 (0.93-1.73) | 1.49 (0.97-2.28) | 0.029 | 1.06 (1.02-1.11) | 0.005 |
| Model 2  OR (95% CI) | 1.00 (Reference) | 1.18 (0.67-2.08) | 1.33 (0.94-1.89) | 1.40 (0.86-2.30) | 0.119 | 1.06 (1.01-1.11) | 0.033 |
| Model 3  OR (95% CI) | 1.00 (Reference) | 1.19 (0.66-2.13) | 1.34 (0.95-1.88) | 1.40 (0.85-2.29) | 0.123 | 1.05 (1.00-1.11) | 0.035 |
| **DSST** |  |  |  |  |  |  |  |
| Case/N | 80/385 | 82/385 | 109/385 | 124/385 |  |  |  |
| Model 1  OR (95% CI) | 1.00 (Reference) | 0.96 (0.67-1.37) | 1.36 (0.87-2.13) | 1.87 (1.17-2.98) | 0.011 | 1.09 (1.02-1.18) | 0.016 |
| Model 2  OR(95% CI) | 1.00 (Reference) | 1.11 (0.73-1.68) | 1.36 (0.82-2.26) | 1.53 (0.98-2.40) | 0.065 | 1.06 (0.99-1.14) | 0.081 |
| Model 3  OR(95% CI) | 1.00 (Reference) | 1.05 (0.67-1.65) | 1.28 (0.73-2.22) | 1.45 (0.93-2.25) | 0.114 | 1.05 (0.98-1.13) | 0.127 |

Model 1: adjusted for age, gender, ethnicity

Model 2: additionally adjusted for income, education, current smoker, current drinker, supplement use, BMI, total intake of energy, AHEI, dietary fiber intake

Model 3: additionally adjusted for self-reported of diabetes, hypertension, cardiovascular diseases (congestive heart failure, coronary heart disease, angina, heart attack, and stroke), cancer, and sleep disorders.

BMI, body mass index; AHEI, alternative healthy eating index; Q, quartile; CERAD-WL, Consortium to Establish a Registry for Alzheimer’s Disease Word List Learning Test; CERAD-DR, CERAD Word List Delayed Recall Test; AF, Animal Fluency; DSST, Digit Symbol Substitution Test.

## Supplementary Figures

**
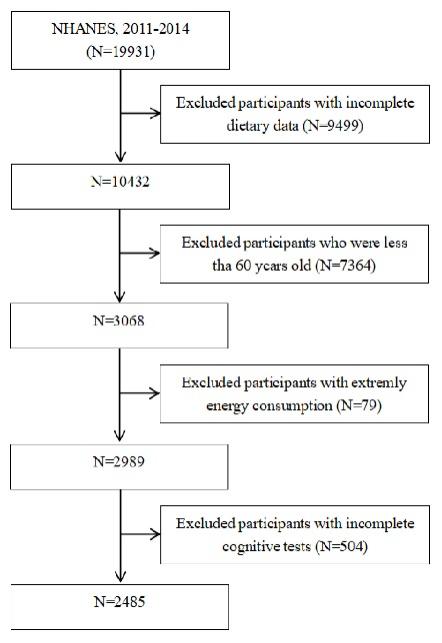
**

**Supplementary Figure 1.** Flow chart of the screening process for the selection of eligible participants
